# Cardiolipin occupancy profiles of YidC paralogs reveal the significance of respective TM2 helix residues in determining paralog-specific phenotypes

**DOI:** 10.3389/fmolb.2023.1264454

**Published:** 2023-10-06

**Authors:** Surabhi Mishra, Evan J. van Aalst, Benjamin J. Wylie, L. Jeannine Brady

**Affiliations:** ^1^ Department of Oral Biology, University of Florida, Gainesville, FL, United States; ^2^ Department of Chemistry and Biochemistry, Texas Tech University, Lubbock, TX, United States

**Keywords:** *Streptococcus mutans*, YidC, cardiolipin, molecular dynamics, paralog

## Abstract

YidC belongs to an evolutionarily conserved family of insertases, YidC/Oxa1/Alb3, in bacteria, mitochondria, and chloroplasts, respectively. Unlike Gram-negative bacteria, Gram-positives including *Streptococcus mutans* harbor two paralogs of YidC. The mechanism for paralog-specific phenotypes of bacterial YidC1 versus YidC2 has been partially attributed to the differences in their cytoplasmic domains. However, we previously identified a W138R gain-of-function mutation in the YidC1 transmembrane helix 2. YidC1^W138R^ mostly phenocopied YidC2, yet the mechanism remained unknown. Primary sequence comparison of streptococcal YidCs led us to identify and mutate the YidC1^W138^ analog, YidC2^S152^ to W/A, which resulted in a loss of YidC2- and acquisition of YidC1-like phenotype. The predicted lipid-facing side chains of YidC1^W138^/YidC2^S152^ led us to propose a role for membrane phospholipids in specific-residue dependent phenotypes of *S. mutans* YidC paralogs. Cardiolipin (CL), a prevalent phospholipid in the *S. mutans* cytoplasmic membrane during acid stress, is encoded by a single gene, *cls*. We show a concerted mechanism for cardiolipin and YidC2 under acid stress based on similarly increased promoter activities and similar elimination phenotypes. Using coarse grain molecular dynamics simulations with the Martini2.2 Forcefield, YidC1 and YidC2 wild-type and mutant interactions with CL were assessed *in silico*. We observed substantially increased CL interaction in dimeric versus monomeric proteins, and variable CL occupancy in YidC1 and YidC2 mutant constructs that mimicked characteristics of the other wild-type paralog. Hence, paralog-specific amino acid- CL interactions contribute to YidC1 and YidC2-associated phenotypes that can be exchanged by point mutation at positions 138 or 152, respectively.

## Introduction

Approximately one-third of all proteins are translocated to various biological membranes to support essential functions such as molecular transport, signaling, and energy transduction. In bacteria, protein translocation to the cytoplasmic membrane occurs through a heterotrimeric proteinaceous channel, SecYEG (Van den Berg et al., 2004). An accessory membrane protein, YidC, functions with SecYEG in the insertion of most of the membrane proteins (Beck et al., 2001; Urbanus et al., 2001; Sachelaru et al., 2017), except for a few cases in which smaller polypeptides with only one or two transmembrane (TM) helices are inserted by YidC in a SecYEG-independent manner (reviewed in (Steinberg et al., 2018)). YidC belongs to the YidC/Oxa1/Alb3 family of proteins that are ubiquitously present in all three domains of life and localized within bacterial, mitochondrial, and chloroplast membranes, respectively (reviewed in (Lewis and Brady, 2015)). Gram-negative bacteria contain a single YidC, whose deletion is lethal, while most Gram-positive bacteria contain two YidC paralogs. These were originally denoted YidC1 and YidC2 in the cariogenic dental pathogen *Streptococcus mutans* (Hasona et al., 2005). The presence of at least one YidC paralog is necessary to support the growth and survival of Gram-positive bacteria; however, different phenotypic consequences are observed upon the elimination of each one suggesting discrete functional activities (Murakami et al., 2002; Tjalsma et al., 2003; Hasona et al., 2005; Dong et al., 2008; Palmer et al., 2012; Palmer et al., 2019; Mishra and Brady, 2021). Paralog-specific protein-protein interactions and individual physiological roles have been identified for YidC homologs of eukaryotic organelles as well (Woolhead et al., 2001; Jia et al., 2003; Fiumera et al., 2007; Benz et al., 2009; Falk et al., 2010). Hence, sequence-structure features that impart distinct functional partitioning of bacterial YidC paralogs are of considerable interest.

**FIGURE 1:**
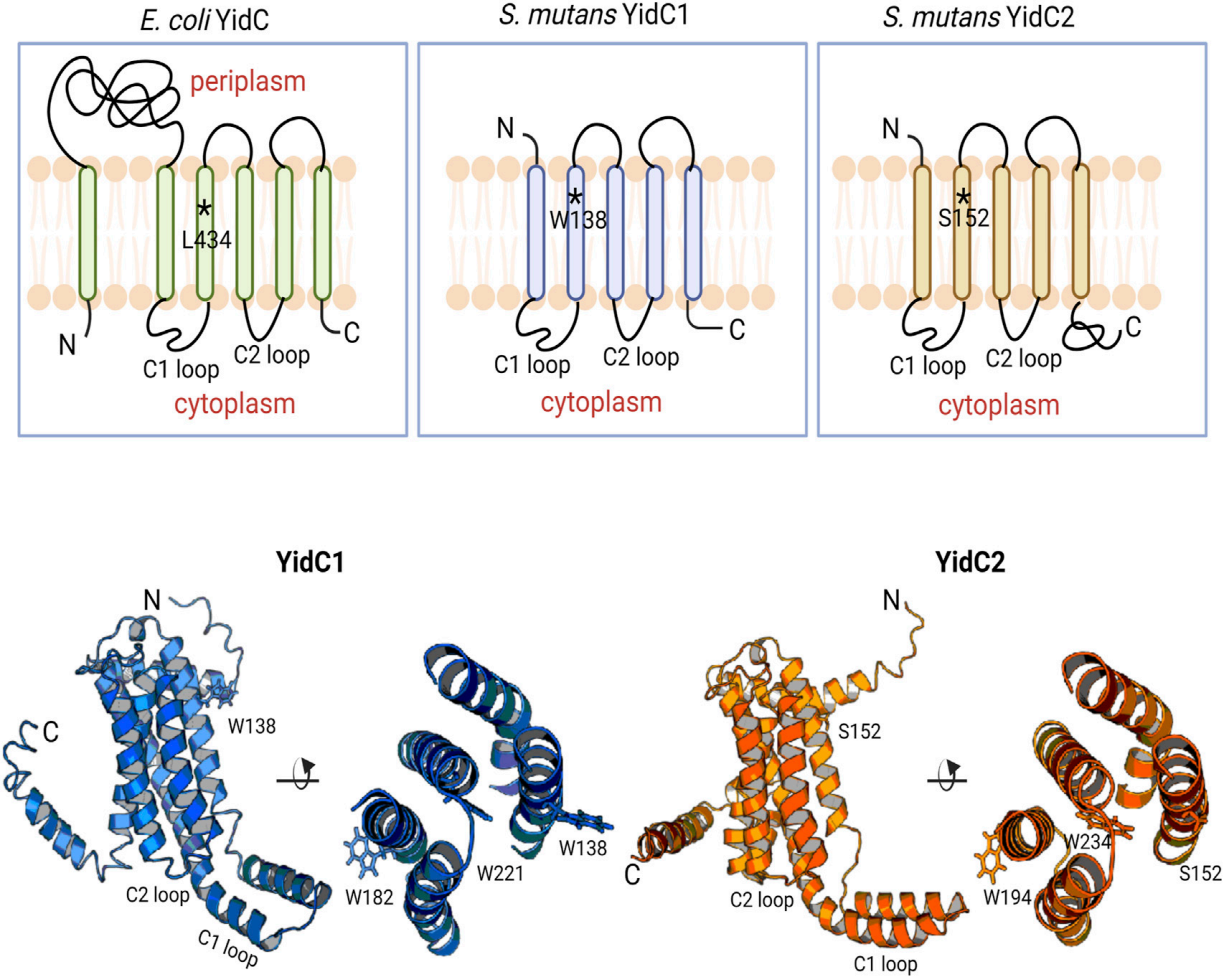
Schematic representation of bacterial YidCs’ topology and structure. Schematic representation of *Escherichia coli* YidC membrane topology compared to *S. mutans* YidC1 and YidC2. Side (left) and top down (right) views of predicted tertiary structures of *S. mutans* YidC1 (blue) and YidC2 (orange). Locations of relevant amino acid residues, and C1 and C2 cytoplasmic loops, are indicated.

Phylogenetic analysis of various *yidC1* and *yidC2* sequences suggests their origin stems from an independent gene duplication event that occurred early during evolution and likely led to the divergent amino acid sequences observed across the spectrum of Gram-positive YidC paralogs (Funes et al., 2009). Despite variation in amino acid sequences, the predicted 3-dimensional (3D) structures of representative Gram-positive YidC1 and YidC2 proteins closely resemble the resolved crystal structures of *Bacillus halodurans* YidC2 and the core domain of *Escherichia coli* YidC (Kumazaki et al., 2014a; Kumazaki et al., 2014b). For all bacterial YidCs, the core domain consists of five TM helices interspersed with two distinct cytoplasmic loops and ending with a C-terminal cytoplasmic tail (Figure 1, top). Notably, YidC2 of Gram-positive bacteria, mitochondrial Oxa1, and chloroplast Alb3 all contain a longer C-terminal tail compared to YidC1, Oxa2, and Alb4, respectively. Previous work from our laboratory showed the functional significance of the longer and more positively charged C-terminal tail of *S. mutans* YidC2 in that partial rescue of the signature acid and salt intolerance characteristic of a *ΔyidC2* mutant was demonstrated following the introduction of chimeric YidC1 in which the YidC2 tail had been appended (Palmer et al., 2012). Furthermore, swapping YidC1 and YidC2 C1 and C2 cytoplasmic loops, singly or in combination with their tails, also revealed contributions to paralog-specific phenotypes (Mishra and Brady, 2021). This is not surprising based on multiple sequence alignment (MSA) of *S. mutans* YidC1 and YidC2 amino acid sequences that reveal that cytoplasmic domains are less well-conserved compared to their respective TM helices (Supplementary Figure S1). Acid sensitivity of the *ΔyidC2* strain was also reversed by a spontaneous YidC1^W138R^ gain of function mutation within the highly conserved TM2 helix (Mishra et al., 2019). This mutation within *yidC1* was reproducibly observed following serial passage of the *ΔyidC2* strain. The underlying mechanism for the intriguing acquisition of YidC2-like behavior by YidC1 following a single point mutation within TM2 led us to explore this question further.

MSA of various Gram-positive YidC1 sequences represented as sequence logo plots further reveals that while TM2 is generally well-conserved, residue W138 itself is not conserved among different species’ YidC1s (Supplementary Figure S2) (Crooks et al., 2004). In contrast, MSA of various Gram-positive YidC2 sequences showed the analogous position within their TM2s to be more highly conserved and usually occupied by serine or methionine (Supplementary Figure S2). This represents S152 in *S. mutans* YidC2, and the analogous position is occupied by serine in other streptococcal and enterococcal YidC2 as well. The analogous residue in *E. coli* YidC is L434. While YidC^L434S^ is able to support the growth of an *E. coli* YidC depletion strain, impaired insertase activity was observed *in vitro* (Jiang et al., 2003). This result further highlights the significance of this specific position on bacterial YidC function. Studies of the abundance of amino acids within TM helices suggest that Trp, Ser, Arg, and Leu are all favored in terms of maintaining helix integrity, although positional conservation can vary due to respective location relative to the bilayer mid-plane (Mbaye et al., 2019).

I-TASSER predicted 3D structures of each *S. mutans* insertase illustrate the potential positional relevance of YidC1 W138 and YidC2 S152 (Figure 1, bottom). Both residues appear to be positioned away from the membrane mid-plane with their side chains facing outward into the lipid bilayer. We therefore hypothesized a contributory role of membrane lipids in YidC1^W138R^ - and YidC2^S152^ - associated growth phenotypes. The lipid composition of the *S. mutans* cytoplasmic membrane was previously characterized by extensive lipidomic analysis carried out in our laboratory (Morales-Aparicio et al., 2020). Cardiolipin (CL) was identified as the predominant phospholipid (60%) in stationary phase cells followed by other anionic phospholipids including phosphatidylinositol (PI), phosphatidylserine (PS), and phosphatidylglycerol (PG). Increased bacterial CL content following acid- or osmotic-stress (Romantsov et al., 2009; MacGilvray et al., 2012; Woodall et al., 2021), as well as increased sensitivity of *S. mutans* lacking *cls*, the sole cardiolipin synthase encoding gene (MacGilvray et al., 2012), have been reported. Although, the mechanisms of CL- and YidC2-dependent protection of *S. mutans* from acid stress are not well understood, the findings that CL and YidC2 are both needed to support *S. mutans* growth at low pH suggests that they may work in concert.

Herein, we demonstrate an increase in *cls* and *yidC2,* but not *yidC1*, promoter activity upon exposure to acid and envelope stress suggesting the coordinate roles of CL and YidC2 in protection from environmental stressors. We also modeled YidC1 and YidC2 interactions with anionic phospholipids, specifically CL, PI, and PS, using a Martini2.2 Forcefield based coarse grain molecular dynamics (CGMD) approach to understand their potential contributions to paralog-specific phenotypic attributes of *S. mutans*. CGMD has proven to be an excellent tool in characterizing protein dynamics (Mustafa et al., 2015; Poma et al., 2017; Damre et al., 2019), oligomerization (Johnston et al., 2012; Prasanna et al., 2014; Pluhackova et al., 2016; Song et al., 2021), and lipid interactions (Ans et al., 2020; Duncan et al., 2020; Yekefallah et al., 2022). Our CGMD analyses demonstrated a preference for dimer formation by both *S. mutans* YidC paralogs, and their substitution variants, and the occupancy map of the YidC1^W138R^ dimer showed a significant gain in CL residency within multiple TM helix residues similar to that observed for the YidC2 dimer. Furthermore, mutation of YidC2 S152 to W not only phenocopied elimination of *yidC2,* but an overall decrease in CL occupancy was observed for the YidC2^S152W^ dimer. These results reveal the positional significance of the respective W138 and S152 TM2 residues in conferring YidC1 versus YidC2 paralog-specific phenotypic properties by virtue of characteristic interactions with a prevalent membrane phospholipid such as CL.

## Results

### Expression of *yidC1^W138R^* phenocopies expression of *yidC2*

**FIGURE 2:**
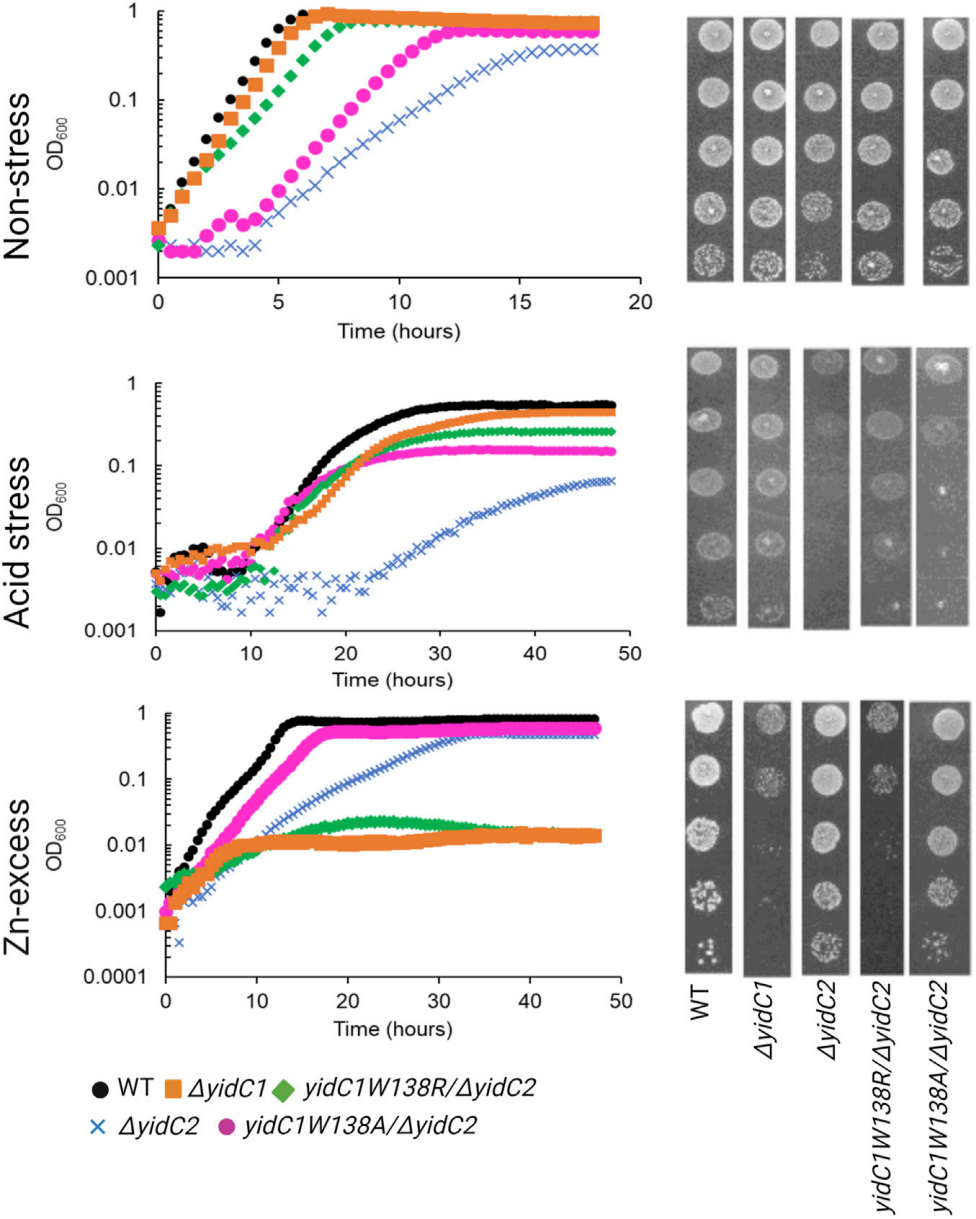
Comparison of growth of *S. mutans* wild-type and mutant strains under non-stress, acid-stress, and zinc-excess conditions. Growth of each indicated strain was monitored under varying environmental conditions in liquid culture (left) and by efficiency of plating on agar (right).

Previous characterization of *S. mutans ΔyidC1* and *ΔyidC2* deletion mutants revealed severe impairment of growth of the *ΔyidC2* strain upon exposure to acid, osmotic, and oxidative stress (Hasona et al., 2005; Hasona et al., 2007; Funes et al., 2009). However, the *ΔyidC2* mutant reproducibly resolved acid-sensitivity by acquiring a point mutation, W138R, in the TM2 domain of YidC1 (Mishra et al., 2019). Unlike deletion of *yidC2*, deletion of *yidC1* does not manifest in sensitivity to environmental stress except under conditions of zinc-excess. Since, *yidC1* ^*W138R*^ expression conferred tolerance to acid-stress in a *ΔyidC2* background, we hypothesized that this strain might show altered sensitivity to Zn-excess. We compared growth of *S. mutans* wild-type (WT), *ΔyidC1*, and *ΔyidC2* strains, as well as strains expressing *yidC1* ^*W138R*^ or *yidC1* ^*W138A*^ in a double *ΔyidC1/yidC2* background, under non-stress, acid stress, and Zn-excess conditions (Figure 2). As expected, deletion of *yidC2* resulted in impaired growth and pronounced acid sensitivity. Growth of the *ΔyidC2* strain under non-stress conditions was restored by expression of *yidC1* ^*W138R*^, and growth at pH 5 was restored by expression of either *yidC1* ^*W138R*^ or *yidC1* ^*W138A*^. Deletion of *yidC1* impacted *S. mutans* growth in THYE containing 2.5 mM ZnCl_2_ far more than deletion of *yidC2,* and zinc tolerance was restored by expression of *yidC1* ^*W138R*^ but not by *yidC1* ^*W138A*^. These results highlight the importance of *S. mutans* YidC1 residue 138 under several biologically relevant environmental conditions and demonstrate that expression of *yidC1* ^*W138R*^ phenocopies expression of *yidC2* when either is present as the sole functional *yidC*.

### CL and YidC2 function in concert to protect *S. mutans* from acid and envelope stress

**FIGURE 3:**
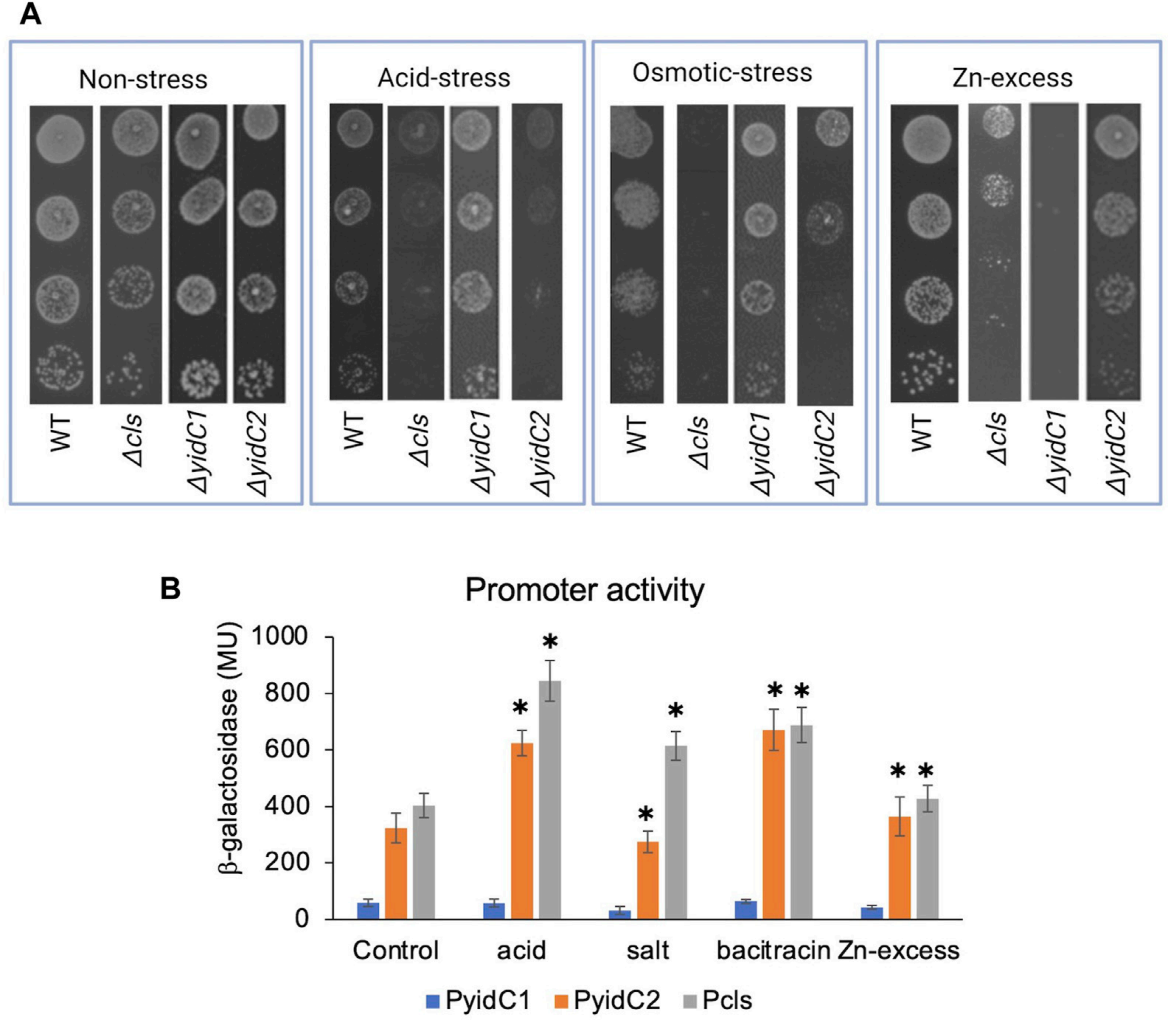
Effect of environmental stressors on growth and promoter activities of *yidC1*, *yidC2*, and *cls*. **(A)** Growth of each indicated strain was monitored under varying environmental by efficiency of plating on agar. **(B)** Promoter activities in mid-exponential phase cultures were measured following 45 min exposure to various environmental stressors. β-galactosidase activity was expressed in Miller Units (MU). The results are representative of three independent experiments performed in duplicate. *Significant difference (*p*-value <0.005), compared to untreated in each case (determined from Student’s t-test).

Often, genes that contribute to the same biological pathways will manifest similar phenotypes upon deletion. To test the degree to which a *S. mutans Δcls* mutant shares known *ΔyidC2-* or *ΔyidC1*-associated properties, WT, *Δcls*, *ΔyidC1*, and *ΔyidC2* strains were compared for their ability to tolerate low pH, high salt, and Zn-excess conditions (Figure 3A). In addition to impaired growth at pH 5, the *Δcls* mutant demonstrated pronounced sensitivity to 3% NaCl, another *ΔyidC2*-associated phenotype. The *Δcls* strain also acquired moderate sensitivity to 2.5 mM ZnCl_2_, but resembled *ΔyidC2* more than *ΔyidC1* in this regard (Figure 3A).

To further evaluate whether YidC2 and CL act in concert to protect against environmental stress, we hypothesized that *yidC2* and *cls* would be transcriptionally upregulated by identical stressors. To test this question, promoter-reporter gene fusions of *yidC1*, *yidC2*, and *cls* were constructed. The genetic context of *yidC1* is well conserved among Gram-positive bacteria with *yidC1* in an operon with the gene encoding the protein subunit of ribonuclease P (*rnpA*). In *S. mutans*, 17 bp of *rnpA*, including the stop codon, overlaps with *yidC1*. The start codon of the downstream gene, *jag*, is located 12 bp from the stop codon of *yidC1*. Bacterial promoter prediction tools (BPROM) identified two distinct −10 and −35 sites upstream of *yidC1* (Solovyev and Salamov, 2011). One set of −10 and −35 boxes is located 256 and 276 bp upstream of *yidC1* and within the *rnpA* gene, while a second set is located 48 and 67 bp upstream of *rnpA*. No transcription termination sites were identified between *rnpA*, *yidC1*, and *jag* suggesting these genes comprise an operon. Based on these predictions, the 470 bp promoter region upstream of the *yidC1* start site was used to construct a *yidC1* promoter-lacZ fusion construct. Unlike *yidC1*, the genomic context of *yidC2* is not conserved. In *S. mutans*, *yidC2* is present 91 bp downstream of an acylphosphatase encoding gene, *acp*, and ∼400 bp upstream of the translation elongation factor, *greA*, which is oriented opposite to *yidC2*. The genetic context of *yidC2* suggests it is likely transcribed from its own promoter. A−10 (TAAAAT) and a −35 (ATTGAGT) box were predicted 40 and 64 bp upstream of the *yidC2* start site, respectively, and a 283 bp segment of the *yidC2* promoter was used to make its *lacZ* promoter-reporter construct. Previous work from the Quivey lab identified the *cls* transcriptional start site 5’ to the upstream gene, *wapA* (MacGilvray et al., 2012). A 300 bp segment upstream of *wapA* was used to make the *cls* promoter-*lacZ* reporter construct. All three promoter-reporter gene fusions were integrated in a single copy at the *phnA-mtlA2* locus within the *S. mutans* UA159 chromosome.

To determine the impact of varying environmental stressors on *yidC1*, *yidC2*, and *cls* expression, β-galactosidase activities were measured in response to acid stress (pH 5), osmotic stress (3% NaCl), Zn-excess (2.5 mM ZnCl_2_), and cell envelop stress (250 μg mL^-1^ bacitracin) conditions and compared with exponential growth-phase expression for each respective promoter (Figure 3B). Acid stress and bacitracin-induced envelope stress led to ∼2-fold induction of *yidC2* and *cls*, while *yidC1* promoter activity remained unchanged under all environmental stress conditions tested. Osmotic stress and Zn-excess had no measurable effect on the activity of any of the promoters. These results are consistent with the hypothesis that YidC2 and CL likely function together to enable acid and envelope stress tolerance. PI and PS biosynthetic pathways have not been characterized in *S. mutans* or other streptococci. Therefore, mutant strains are not available to explore potential phenotypic overlap between *ΔyidC1* and *ΔyidC2* strains and mutants lacking anionic phospholipids other than CL.

### YidC1 and YidC2 likely function as dimers in a CL-rich membrane

Due to challenges in adequate expression and purification of recombinant YidC1 and YidC2 proteins in sufficient quantity for lipid-protein interaction studies, we used coarse grain molecular dynamics (CGMD) simulations to study the impact of membrane lipids on *S. mutans* YidC paralogs in our current study. CL, PI, and PS together comprised ∼90% of the total phospholipid content of cytoplasmic membrane samples prepared from *S. mutans* cells harvested from stationary phase cultures (Morales-Aparicio et al., 2020). CL, PI, and PS are anionic phospholipids with negatively charged polar head groups (Supplementary Figure S3). We modeled CL, PI, and PS at a 3:1:1 molar ratio mimicking the *S. mutans* lipid bilayer. To start, monomeric YidC1 or YidC2 simulations containing the CL-rich tri-lipid mixture were analyzed to identify potential interactions between monomeric forms of YidC1 and YidC2 with the membrane lipids. A potential monomeric organization of YidC1 and YidC2 was evaluated first based on crystallographic data of various YidCs from *B. halodurans* (Kumazaki et al., 2014a; Kumazaki et al., 2014c), *E. coli* (Kumazaki et al., 2014b), and *Thermotoga maritima* (Xin et al., 2018; Nass et al., 2022). Here, and for simulations described below, single systems were built using the CHARMM-GUI (Jo et al., 2008; Lee et al., 2016) Martini maker (Qi et al., 2015; Hsu et al., 2017) and were energy minimized and equilibrated in the Groningen Machine for Chemical Simulation (GROMACS) (Abraham et al., 2015). After equilibration, systems were cloned into 5 replicates (Knapp et al., 2018) to ensure equivalent starting energy profiles, and production was carried out for a total of 10 μs per simulation. The average RMSD of Cα-atoms provides insight into system stability for a given protein throughout the simulation. All systems that we assessed were observed to quickly reach equilibrium within ∼2 Å Cα RMSD compared to the final snapshot of respective equilibration steps (Supplementary Figure S4). PyLipID was then used to calculate lipid residency times by residue (Song et al., 2022). Lastly, we calculated the RMSF values for each amino acid residue during the course of the simulation of YidC1 and YidC2 in the 3CL:PI:PS mixture to understand environment-dependent protein dynamics (Supplementary Figure S5). RMSF values represent the extent of fluctuation for each residue around their average position. Interestingly, none of the YidC1 or YidC2 residues were significantly occupied (>3 μs) by CL in the simulated CL-rich bilayer (Supplementary Figure S6). Average RMSF values of residues comprising the cytoplasmic loops as well as the TM helices were less than 2 Å in both YidC1 and YidC2 monomers suggesting relatively inflexible structures. Collectively, our results support the notion that monomeric YidC1 and YidC2 do not represent the functional forms of the insertases *in vivo*. This observation is predicated upon the expectation that insertases exist *in vivo* as oligomers or in heteromeric complexes. Since CL interaction is likely related to function, and significant CL interaction is observed only for dimer simulations (discussed below), we conclude that insertase monomers are likely not functionally relevant units in a CL-rich environment.

We next tested whether the assembly of YidC1 or YidC2 as dimers within the simulated bilayer would change the potential lipid-interacting interfaces of the proteins. A dimeric YidC structure has been proposed in the literature based on bioinformatic analysis of an ancestral YidC-like protein as the progenitor of SecY (Lewis and Hegde, 2021), as well as experimental data on *E. coli* YidC and other Oxa1 family proteins, EMC3 and GET1 (Oliver and Paetzel, 2008; Ravaud et al., 2008; McDowell et al., 2020; O'Donnell et al., 2020; Pleiner et al., 2020). Recently a preprint supporting a functional dimeric pore-like structure of *E. coli* YidC, based on fluorescence correlation spectroscopy, Blue-Native PAGE, reconstitution of purified protein into vesicles, and single molecule microscopy, was published (Knyazev et al., 2023).

**FIGURE 4:**
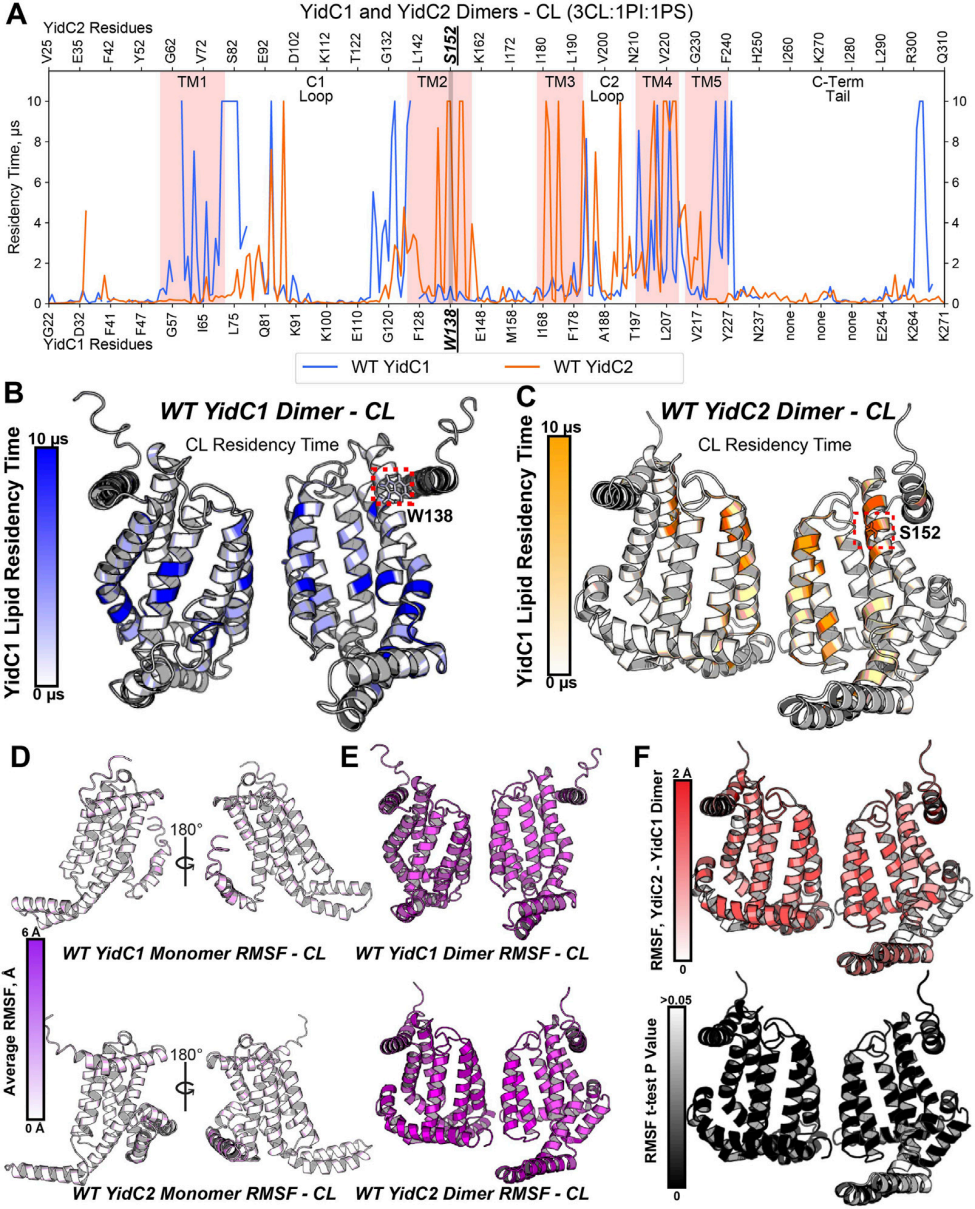
Phospholipid residency and molecular dynamics (RMSF) derived by coarse grain molecular dynamics (CGMD) analysis of YidC1 or YidC2 in simulated cardiolipin (CL)-rich bilayer. **(A)** Phospholipid residency plot of YidC1 and YidC2 dimer in CL-rich bilayer. Residency times of individual numbered residues of YidC1 (denoted at the bottom of each graph) or YidC2 (denoted at the top of each graph) are shown. Results indicate occupancy of CL when simulations were performed in a CL-rich (3CL:1PI:1PS). Pink shading indicates residues comprising transmembrane domains 1 through 5. Locations of the C1 and C2 cytoplasmic loops and the C-terminal cytoplasmic tail segments are also indicated. **(B)** CL residency plotted as heat map on YidC1 dimer 3D model structure, **(C)** CL residency plotted as heat map on YidC2 dimer 3D model structure. The intensity of color deepens with an increase in residency times. Dynamics of YidC1 and YidC2 (RMSF values) plotted as heat maps on modeled structure: **(D)** monomers, and **(E)** dimers. **(F)** *p*-values per residue of the RMSF differences between YidC2 and YidC1 dimers. T-test analyses were performed comparing RMSF values per residue from each simulation replicate of dimeric YidC2 to values derived from YidC1 simulations.

To model how dimeric YidC1 or YidC2 interact with membrane phospholipids, simulation systems containing the CL-rich tri-lipid mixtures were built around constructed homodimers of WT YidC1 and WT YidC2. Notably, the TM4 helices of *S. mutans* both YidC1 and YidC2 align with TM5 of *E. coli* YidC, which was proposed by Lewis and Hegde to be present at the dimer interface (Lewis and Hegde, 2021). Thus, dimer structures were built by cloning and placing a 180-degree rotated monomer respective to the original monomer with TM4-TM4 distance measured to be 7–8 Å using PyMol. Dimeric simulations were energy-minimized and equilibrated identically to monomeric simulations, and production was carried out for 10 μs prior to trajectory analysis. As with monomeric simulations, dimeric YidC1 and YidC2 displayed average RMSD values less than ∼2 Å for Cα-atoms when fit to the final equilibration frame of respective equilibration runs (Supplementary Figure S4). High CL densities were observed around multiple residues for both YidC1 and YidC2 dimeric structures (Figures 4A–C). Although the number of CL-occupying residues was comparable for YidC1 and YidC2, 38 and 32, respectively, CL occupied distinctly different locations within each dimer. Residues within TM1, TM4 and TM5 helices were strongly occupied by CL in dimeric YidC1 in comparison to multiple residues within TM2, TM3, and TM4 helices in dimeric YidC2. Notably, CL significantly occupied the TM4-TM4 dimer interface in both YidC1 and YidC2. In our simulations, cytoplasmic loop 1 residues at the junctions of TM1 and TM2 were significantly occupied by CL in the YidC1 dimer, but those sites were unoccupied in the YidC2 dimer. A lack of CL occupancy at the lipid-water interface in the TM1 and TM2 helices of YidC2 suggests a more flexible C1 loop in YidC2 compared to YidC1, which is supported by RMSF analysis (Supplementary Figure S5). In addition, RMSF analyses suggest structural plasticity in dimeric structures for both YidC1 and YidC2 when compared to their monomeric forms (Figures 4D, E). The coincident observations of increased dynamics and enhanced CL interaction as dimers is intriguing.

When assembled as dimeric forms in the CL-rich environment, differences in RMSF values between YidC1 and YidC2 were quite substantial. This information is illustrated by a YidC2 minus YidC1 heat map (Figure 4F). The YidC2 dimer appears more dynamic by approximately 1 Å compared to YidC1 for each aligned residue pair. Although a 1 Å RMSF difference in magnitude at a given individual position may not seem high, statistically significant differences (*p*<0.05) were observed for nearly every homologous residue. Collectively, these results reveal a level of structural flexibility for the YidC2 dimer in a CL-rich environment that is not as strongly shared by the YidC1 dimer. In addition, both YidC1 and YidC2 dimers acquired substantially increased CL occupancies compared to their respective simulated monomers, and the patterns of CL occupancy varied between the paralogs. A dimer-promoting role of CL was previously reported for SecYEG (Corey et al., 2018); therefore, it is not surprising that YidC1 and YidC2 both formed stable dimers in the simulated CL-rich environment. None of the YidC1 dimer residues showed significant PI or PS occupancy in the CL-rich tri-lipid mixture (Supplementary Figure S7). In contrast, the YidC2 dimer showed significant residency times with all three anionic phospholipids in the simulated bilayer (Supplementary Figure S7).

### YidC1^W138R^ and YidC2 dimers are similar in their interactions with CL

**FIGURE 5:**
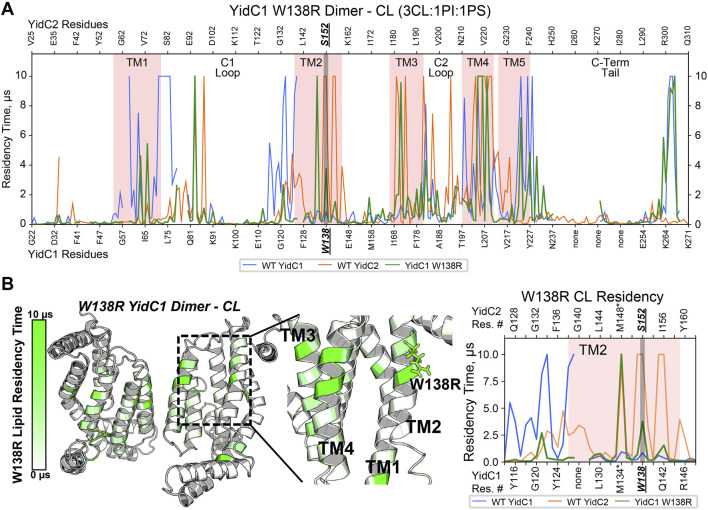
Comparison of CL occupancy of YidC1^W138R^ dimer versus YidC1 and YidC2 dimers derived from CGMD in a simulated CL-rich bilayer. **(A)** Residency plot. Residency times of individual numbered residues of YidC1^W138R^ (denoted at the bottom of each graph) or YidC2 (denoted at the top of each graph) are shown. Pink shading indicates residues comprising transmembrane domains 1 through 5. Locations of the C1 and C2 cytoplasmic loops and the C-terminal cytoplasmic tail segments are also indicated. **(B)** Heat map of CL residency of YidC1^W138R^ dimer. Sites close to W138R mutation were zoomed in the heat map and residency plot.

To understand how YidC1^W138R^ might impact lipid interaction and protein dynamics, we also simulated this mutated protein in a CL-rich environment for 10 μs per replicate. Structure files were prepared by introducing the point mutation to the WT, membrane-aligned, YidC1 monomer or dimer model prior to system generation in CHARMM-GUI. When evaluated in either monomeric or dimeric forms in the simulated CL-rich bilayer, YidC1^W138R^ became stable in <1 µs (Supplementary Figure S4). RMSF analysis and residue-wise comparison with the WT YidC1 dimer did not identify significant differences suggesting that the W138R point mutation did confer an overall dynamic structure like that observed for the YidC2 dimer. Both YidC1 and YidC1^W138R^ dimers were less dynamic than the YidC2 dimer in the CL-rich environment (Supplementary Figure S5). Nevertheless, the W138R point mutation within TM2 of YidC1 resulted in a loss of CL interactions with multiple residues within TM1, and TM1-C1 loop and TM2-C1 loop junctions (Figure 5A). Lipid occupancy maps further showed gain of CL interactions with residues within TM2 and TM3, a feature characteristic of the YidC2 dimer (Figures 5A, B). Because it is negatively charged, CL would be expected to interact better with positively charged Arg, compared to hydrophobic Trp. This was evidenced by the increased CL residency time at R138 of the mutant YidC1 dimer compared to W138 of the wild-type YidC1 dimer. Notably, the lipid occupancy map of the YidC1^W138R^ dimer acquired multiple similarities to that of the YidC2 dimer except at the C-terminal tail, which more closely resembled the YidC1 dimer (Figure 5A). Neither PI nor PS showed any significant occupancy with the YidC1^W138R^ dimer (Supplementary Figure S8). Collectively, evaluation of lipid interactions with the varying YidC monomers and dimers reveals the significance of CL in the YidC1^W138R^ acquisition of YidC2-like features, namely a gain in ability to support acid tolerance and a loss in tolerance to Zn-excess. Similar to monomeric YidC1 and YidC2, the YidC1^W138R^ monomer likewise did not demonstrate significant lipid occupancies (Supplementary Figure S6), further supporting a dimeric structure as the functionally relevant form of YidC insertase paralogs.

### Impact of YidC2 S152 point mutations on *S. mutans* phenotypic properties and modeled CGMD interactions with specific lipids

**FIGURE 6:**
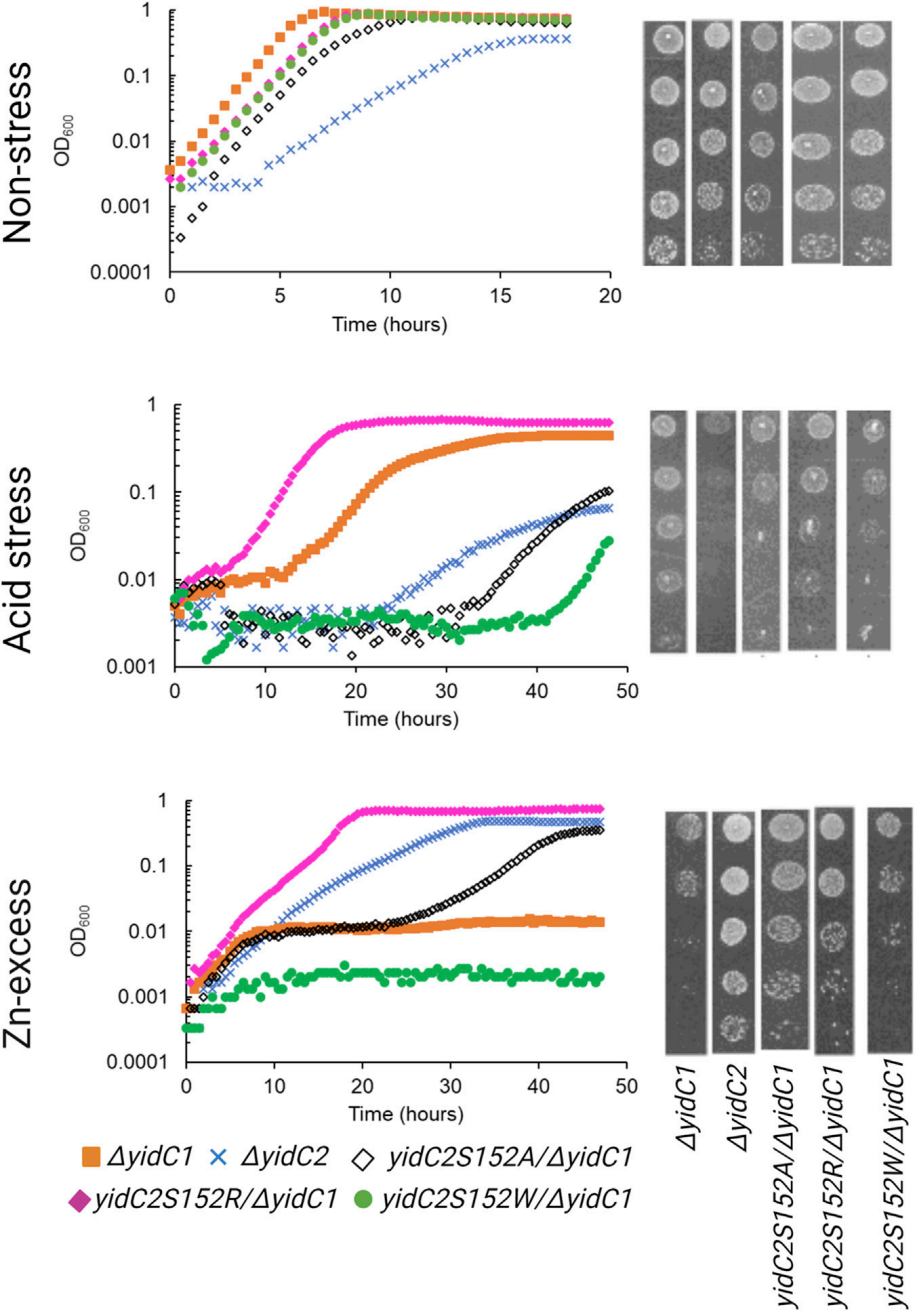
Comparison of growth of *S. mutans* YidC2 mutant strains with YidC1 and YidC2 harboring strains under non-stress, acid-stress, and zinc-excess conditions. Growth of each indicated strain was monitored under varying environmental conditions in liquid culture (left) and by the efficiency of plating on agar (right).

The acquisition of YidC2-associated behaviors by the YidC1^W138R^ mutant prompted us to also engineer substitution mutations at position 152 of YidC2. These included S152W, S152A, and S152R mutations, with each gene variant produced as the sole *yidC* paralog in a *ΔyidC1/yidC2* background. First, we assayed each strain for growth under non-stress conditions (Figure 6). As expected, the *ΔyidC2* strain demonstrated an obvious growth defect compared to the *ΔyidC1* strain. None of the amino acid substitutions at YidC2 position 152 conferred a severe growth defect of *S. mutans* in liquid culture, although growth of the YidC2^S152A^ variant was somewhat more impacted than the YidC2^S152R^ and YidC2^S152W^ strains. Similar results were observed in the efficiency of plating assays. Importantly, when *S. mutans* growth was evaluated at pH 5, the YidC2^S152A^ and YidC2^S152W^ strains were both acid sensitive, as was the *ΔyidC2* deletion mutant (Figure 6). This indicates that neither YidC1, nor YidC2^S152A^ or YidC2^S152W^ variants, can efficiently insert those substrates necessary for acid tolerance in *S. mutans*. In stark contrast, the YidC2^S152R^ strain tolerated acidic pH even better than the *ΔyidC1* strain that produces unmodified YidC2. That is, YidC2-mediated acid tolerance was improved by the YidC2^S152R^ substitution. Again, results were recapitulated in efficiency of plating assays. Collectively, these results demonstrate the critical importance of residues 138 and 152 to YidC1 and YidC2s’ respective abilities to support acid tolerance, or not. Taken together, our results reveal that a Trp at this position is associated with acid sensitivity, while a Ser or Arg at this position enables the insertase to support acid tolerance as is observed for unmodified YidC2, YidC2^S152R^, and YidC1^W138R^.

Lastly, *S. mutans* was grown in the presence of excess Zn (Figure 6). As expected, growth of the *ΔyidC1* mutant was severely impaired in liquid culture and on agar plates. The YidC2^S152W^ strain also acquired the severe *ΔyidC1*-associated zinc sensitivity phenotype. In other words, the YidC2^S152W^ variant became YidC1-like. In addition, the YidC2^S152A^ strain also became intolerant to excess zinc, though more so in liquid culture than on agar plates. Again, the result of the YidC2^S152R^ mutation was opposite to that of the other amino acid substitutions tested. In this case, zinc tolerance was notably improved in liquid culture. When tested on agar plates, the YidC2^S152W^ mutation was as severe as eliminating *yidC1,* but less impact was observed for YidC2^S152R^ followed by YidC2^S152A^ in efficiency of plating assays. Lack of complete concordance of results in liquid culture compared to those on agar plates could stem from differences in insertion efficiency or functionality of relevant substrates under conditions of varying aeration, or as yet unknown changes in lipid composition in the presence of excess zinc.

**FIGURE 7:**
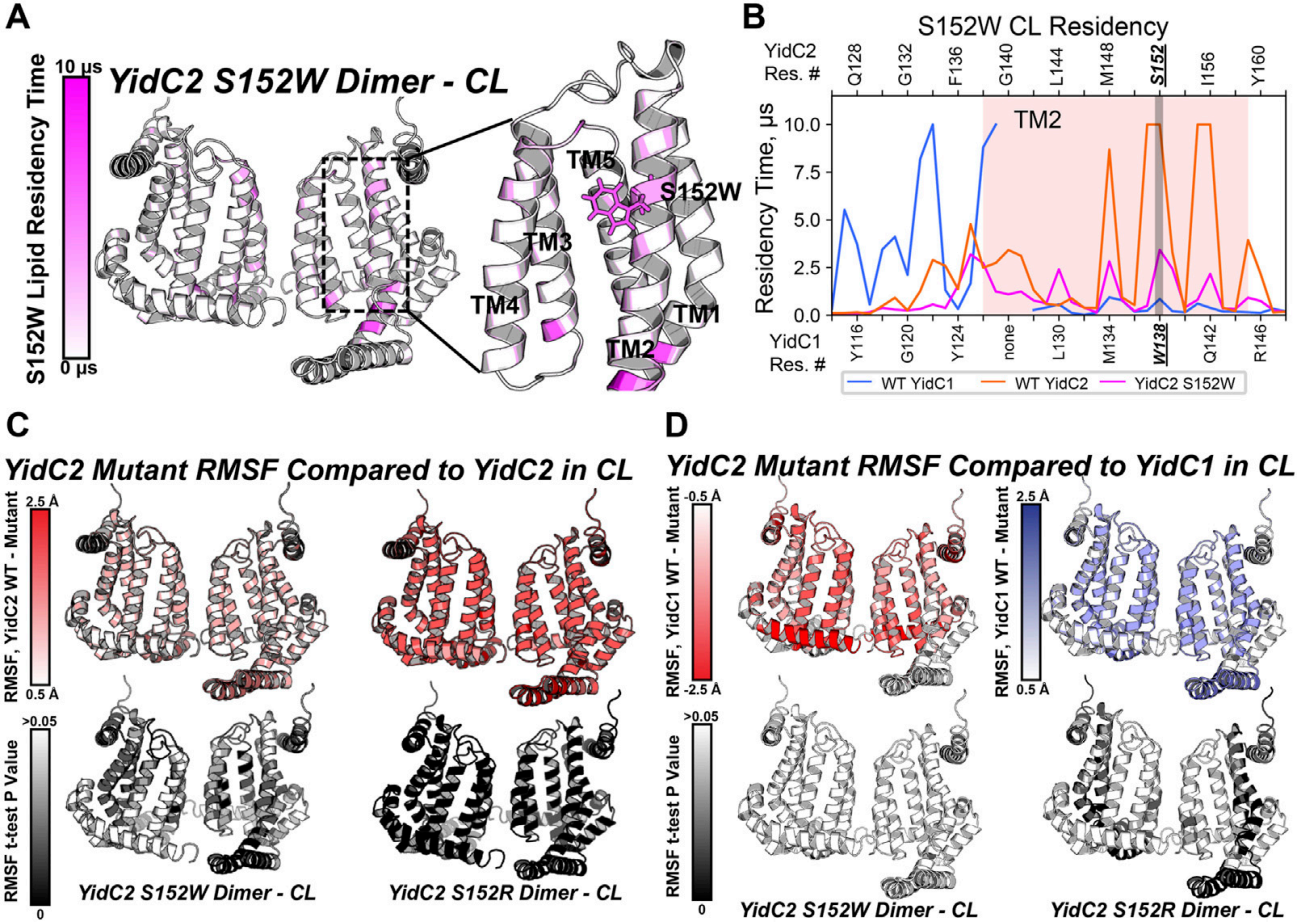
Comparison of mutant YidC2 CL interactions and dynamics, versus those of YidC1 and YidC2 dimers derived by CGMD in a simulated CL-rich bilayer. **(A)** Heat map showing CL occupancy of the YidC2^S152W^ mutant zoomed in on the S152W region. **(B)** CL residency of TM2 helix residues presented as a plot illustrating that S152W severely reduced CL interaction at position 152 in YidC2. Dynamics of YidC2 mutants determined from RMSF values compared to WT YidC2 **(C)** and YidC1 **(D)** dimers, presented as heat maps (top). *p*-values of the RMSF differences per residue, compared to dimeric WT YidC2 (left) and YidC1 (right), are plotted as heat maps onto respective model structures. T-test analysis was performed comparing RMSF values per residue from each simulation replicate of respective datasets.

Because the *yidC2* ^*S152W*^-expressing strain phenotypically resembled the strain expressing *yidC1* only, we wanted to understand the impact of the S152W mutation on phospholipid interactions at the level of individual residues in comparison to dimeric unmodified YidC1 and YidC2. As with dimeric YidC1^W138R^ simulations, YidC2^S152W/R^ structure files were prepared by introducing the point mutation to the WT, membrane-aligned, YidC2 dimer model prior to system generation and production MD in the simulated CL/PI/PS tri-lipid bilayer. RMSD values equilibrated to <2.5 Å compared to the final snapshot of equilibration, indicating system stability (Supplementary Figure S4). After PyLipID analysis, the CL occupancy profile of YidC2^S152W^ dimers clearly showed decreased intensity at all positions, including S152, compared to YidC1 or YidC2 dimers (Figures 7A, B; Supplementary Figure S9). The most apparent CL occupancy observed for the YidC2^S152W^ dimer was at residues within the cytoplasmic C1 loop close to the TM1 interface. This would be expected to confer rigidity to the C1 loop region similar to that observed for the YidC1 dimer. PI occupancy for the YidC2^S152W^ dimer followed a pattern generally similar to that of CL occupancy; however, PI occupancy was gained for residues within the C-terminal tail of the YidC2^S152W^ mutant dimer (Supplementary Figure S9). Similar to results observed for CL occupancy, the YidC2^S152W^ mutation also resulted in an overall loss of PS occupancy at numerous residues within the YidC2^S152W^ dimer. This distinguished it from the unmodified YidC2 dimer with regard to phospholipid residency (Supplementary Figure S7). Comparison of RMSF values of YidC2^S152W^ residues with those of the YidC2 dimer showed a ∼0.5–1 Å decrease in the RMSF values for approximately 2/3 of YidC2^S152W^ residues (Figure 7C, left). Although these decreases in RMSF values in YidC2^S152W^ were small compared to those in unmodified YidC2, they were statistically significant as determined by t-test indicating the S152W mutation resulted in a global decrease in YidC2 protein dynamics (Figure 7C, left, Supplementary Figure S5).

Phenotypic characterization of the panel of YidC2 point mutants showed that substitution of Arg for Ser at position 152 favored growth under all conditions tested. Similar to every YidC variant tested, YidC2^S152R^ quickly equilibrated within the CL-rich membrane, and the CL occupancy profile of the YidC2^S152R^ dimer was similar to that observed for the WT YidC2 dimer (Supplementary Figures S7, S9). However, S152R showed a lower CL residency time compared to S152. Residue-based RMSF analysis suggests an overall decrease in the dynamics of YidC2^S152R^ in a CL-rich environment compared to the results for unmodified YidC2 (Figure 7C, right). Interestingly, YidC2^S152W^ RMSF values compared to those of WT dimeric YidC1 are not statistically significant, which is taken as further supportive evidence that YidC2^S152W^ phenocopies WT YidC1 (Figure 7D, left). In contrast, RMSF values of the YidC2^S152R^ dimer suggest increased dynamics compared to unmodified dimeric YidC1 (Figure 7D, right). That is to say, the YidC2^S152W^ dynamic profile was more similar to that of YidC1 than to that of WT YidC2, and the YidC2^S152R^ dynamic profile was statistically significantly different from either WT YidC1 or WT YidC2. The latter observation was somewhat surprising since in other cases we were able to relate dynamic behavior observed in CGMD experiments to improved YidC2 functionality *in vivo*. An important caveat, however, is that our simulations were carried out in the absence of a confirmed substrate. Respective substrates of bacterial translocation systems, of which there are potentially hundreds, are largely unknown. Even in the most widely studied organism *E. coli,* only a handful of membrane-localized substrates are verified and utilized in model experimental systems.

## Discussion

Although initially evolved by gene duplication events, paralogous proteins achieve distinct physiological roles due to differences in amino acid sequences, cellular localization, and genetic regulation. In this study, we identified distinct phospholipid-protein interaction profiles as another feature that delineates paralogous proteins’ functions in a case in which they are membrane localized. Paralogs of membrane proteins, though less common than their cytoplasmic counterparts, include many relevant examples in bacteria such as SecY/SecY2, MpfA/MpfA2, and various DedA paralogs. These paralogs offer interesting examples for further investigation in this regard.

In this study, we showed that *S. mutans* YidC paralogs demonstrate distinctly different CL residency profiles despite remarkable similarity in their 3D structures. Phospholipid-protein interactions are known to modulate spatial localization, oligomerization, and protein-protein interactions within multiprotein complexes (reviewed in (Corradi et al., 2018)). Hence, we postulate that differences in *S. mutans* YidC1 and YidC2 lipid occupancy can lead to distinct protein-protein interactions and oligomeric status thereby contributing to their distinct phenotypic properties. We identified a specific residue within the TM2 domain of YidC1 and YidC2, W138/S152, which significantly influences CL residency across all residues in YidC1 and YidC2 dimers in a Cl-rich bilayer. To the best of our knowledge, these results represent the first report of the positional significance of a single TM domain residue that is significantly responsible for paralogous membrane protein phenotypes, and that when appropriately altered mimics the reciprocal paralog by modulating phospholipid residency profiles.

To date the functional oligomeric status of the most widely studied bacterial YidC, that from *E. coli*, is still debated. Early reports predicted a dimeric structure (Lotz et al., 2008; Kohler et al., 2009); however, crystal structure proposed a monomeric organization for both *E. coli* and *Bacillus halodurance* YidC (Kumazaki et al., 2014a; Kumazaki et al., 2014b). In an interesting insight on the evolution of SecY, YidC was predicted as a dimer (Lewis and Hegde, 2021). Lastly, a recent manuscript preprint reported dimeric assembly of an *E. coli* YidC ion-conducting pore (Knyazev et al., 2023). Because their functional oligomeric status in the *S. mutans* cytoplasmic membrane is unknown, we modeled YidC1 and YidC2, and their substitution derivatives, both as monomers and as dimers. Although monomeric and dimeric forms of both paralogs were stable within the CL-rich tri-lipid environment modeled in our studies, stronger affinity with anionic phospholipids was observed for dimers compared to monomers in all cases. Approximately 10%–15% of residues were occupied by CL in both YidC1 and YidC2 dimers, in contrast to nearly none in their monomeric counterparts. The potential significance of CL-membrane protein interactions in the stabilization of dimeric structures has been reported for the SecYEG translocon, the Na^+^/H^+^ antiporter NhaA, and the multidrug efflux pump EmrE (reviewed in (Corradi et al., 2018)). It is highly likely, therefore, that *S. mutans* YidC1 and YidC2 also function as dimers within a CL-rich membrane.

Several studies using human glycophorin A (GpA) as a model protein have shown that a GxxxG motif within integral membrane protein TMs often leads to dimerization (Brosig and Langosch, 1998). An important feature of GxxxG, or similar AxxxG or SxxxG motifs, is the formation of interhelical carbon-hydrogen bonds that occur across helix-helix interfaces between Cα-H donors and backbone carbonyl (O=C) acceptors (Senes et al., 2000). The motif’s xxx residues can be occupied by 9 amino acids: Gly, Ala, Ser, Thr, Val, Leu, Iso, and Pro. These amino acids are preferred because each contains small side chains thereby avoiding steric hindrance and allowing protein backbone residues to be close enough for a helix-helix interaction to occur. A GxxxG motif (GAPAG) occurs within the TM4 helix of YidC2, and appears to be located at the dimer interface within the simulated CL-rich membrane.

The preference of *S. mutans* YidC2 in particular for dimer formation was also evident based on our observation of the dynamic behavior of YidC2 within the simulated CL-rich bilayer. Crystal structures of *B. halodurans* YidC2 and *E. coli* YidC have suggested that their C1 loops represent dynamic segments that are likely to be involved in substrate identification (Kumazaki et al., 2014a; Kumazaki et al., 2014b; Kumazaki et al., 2014c). When the *E. coli* YidC crystal structure (PDB ID: 3WVF) (Kumazaki et al., 2014b) was modeled in a simulated POPE:POPG (3:1) bilayer, compaction of the protein structure and bilayer thinning was observed in the vicinity of the YidC molecule (Kumazaki et al., 2014b; Chen et al., 2017). The structural compaction of *E. coli* YidC was proposed to stem from an interaction between its C1 loop residues and the hydrophilic head groups of PE and PG, thereby bringing the C1 loop closer to the membrane (Chen et al., 2017). Thus, the presence of lipids appeared to render the *E. coli* YidC C1 loop less flexible compared to that observed within crystal structures that were determined in the absence of lipids. In another instance, a cryo-EM structure of the *E. coli* YidC-ribosome complex within DPPG/DPPC liposomes suggested large conformational changes within YidC’s membrane-embedded core, especially within the TM2 and TM3 helices, which were connected by the C1 loop and extramembrane domain N-terminal to the TM2 helix (Kedrov et al., 2016). The structural flexibility of membrane-localized protein transport machinery components becomes more apparent in the presence of their cognate substrates. In fact, MD simulation of *E. coli* YidC-mediated insertion of the Pf3 coat protein predicted a conformational change within the insertase’s structure during substrate insertion consistent with a requirement for flexibility (Polasa et al., 2022). CGMD analysis of the *S. mutans* YidC1^W138R^ dimer in a simulated CL-rich bilayer resembled that of the YidC2 dimer with regard to a lack of CL-TM1 and CL-C1 loop contacts, as well as gain of CL-contacts in TM3 and TM4 helices. This configuration may enable conformational flexibility of the cytoplasmic domains of the YidC1^W138R^ dimer in a CL-rich environment, although this was not readily apparent in RMSF calculations. As stated, our simulations were performed in the absence of substrates. We propose to confirm the dynamic behavior of YidC1^W138R^ and YidC2 dimers in future studies once YidC2-specific substrates are identified.

**FIGURE 8:**
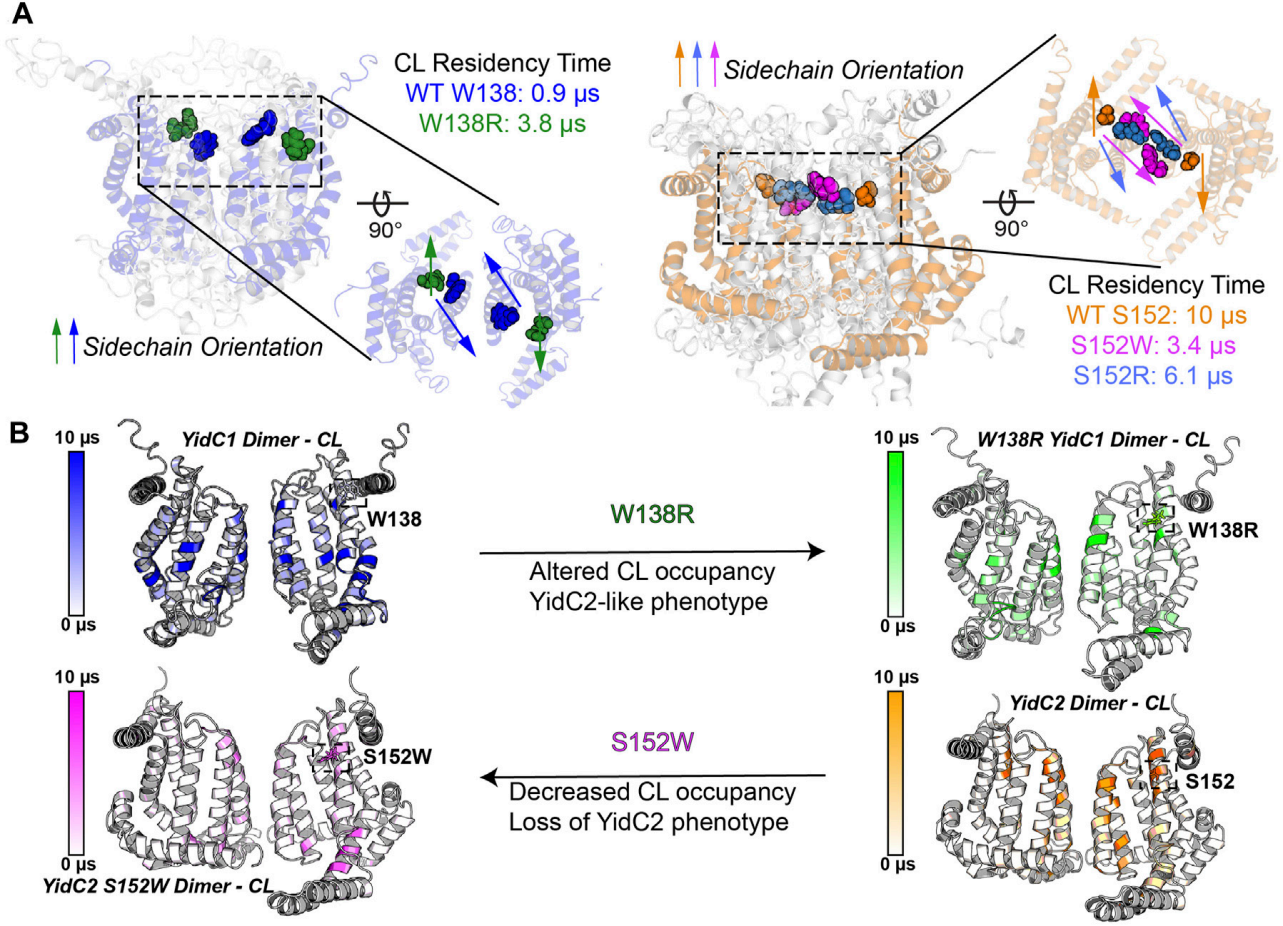
Schematic representation of the impact of YidC1 and YidC2 residues 138 and 152 on cardiolipin occupancy. **(A)** Sidechain orientation of indicated residues in the final CGMD trajectory frames is loosely predictive of CL residency time. Side and top-down views of YidC1 (left) and YidC2 (right) are illustrated. **(B)** Heat maps of CL occupancy derived by CGMD of YidC1 and YidC2 dimers compared to the corresponding W138R and S152W variants are shown. YidC1 W138R acquires YidC2-like properties, while YidC2 S152W loses CL occupancy and YidC2-associated functional activities.

Trp, Ser, Ala, and Arg are all favored residues within TM helices (Liu et al., 2002). Therefore, YidC1’s W138R or YidC2’s152W/R mutations would not be expected to result in large scale changes in protein structure. However, a Trp residue’s location at the water-lipid interface, such as YidC1 W138 or modified YidC2 W152, could impart both aromatic-lipid acyl chain hydrophobic interactions and indole N-lipid headgroup hydrogen bonding. Serine is a non-charged amino acid with a polar side chain, which is favored in interfacial regions, forming hydrogen bonds with other polar residues within TM helices or with polar head groups of membrane phospholipids (Li et al., 2012). The large side chain of Arg is positively charged, and its orientation within a TM helix can allow formation of salt-bridges with negatively charged lipid head groups or with negatively charged amino acids. Thus, Trp, Ser, and Arg have unique lipid-interaction profiles that are dependent on their locations within a given protein present in a lipid bilayer and would be expected to modulate the structure-function of membrane proteins like *S. mutans* YidC1 and YidC2. In CGMD simulations of dimeric YidC variants, YidC1 W138 demonstrated little CL residency (0.9 µs), which increased to 3.8 μs upon mutation to R138. Conversely, YidC2 S152 CL residency (10 μs) was reduced by mutation to R (6.1 μs), and almost abolished by mutation to W (0.7 μs). As summarized in Figures 8A, B, YidC2 or YidC1 dimeric forms demonstrate substantial CL residency when the residue at position 152 or 138, respectively, is Ser or to a lesser extent Arg, but not when the residue is Trp. Orientation of these residues relative to the dimer interface may also contribute to CL residency time (Supplementary Figures S7–S9). It is likely that YidC1 W138 and YidC2 S152W residues are embedded and contact at the dimer interface, rather than extending into the bilayer, thereby mechanically shielding these residues from lipid residency. YidC2 152R/S, and to a lesser extent YidC1 138R/W, appear partially exposed, while YidC2 S152 is generally unencumbered giving rise to CL interaction throughout the entire simulation trajectory.

Notably, Ser, Leu, and Arg are each encoded by six different genetic codons, the maximum observed for any amino acid. Trp is encoded by a single non-redundant genetic codon, TGG. Hence, any mutation to Trp, or from Trp to Ser/Arg/Leu, would be expected to have a substantial impact on the structure-function of a protein. Trp residues are often found in amphipathic helices thought to “slide” along the membrane during functional cycles of membrane proteins. Interestingly, multiple Trp residues (4–6) are common among Gram-positive YidC1 sequences, whereas YidC2 sequences contain only 2-3 Trp residues. *S. mutans* YidC1 contains five Trp residues (W34, W138, W157, W182, and W221), while YidC2 contains three (W37, W194, and W234). Of these, W34/37, W182/194, and W221/234 are conserved between the YidC1 and YidC2 paralogs, respectively. All Gram-positive YidCs contain W221 or W234 in TM5, which fall within the hydrophilic groove of YidC1 and YidC2, respectively. The analogous Tyr residue in *E. coli* YidC has been shown to be important for insertase activity (Chen et al., 2022). In contrast, W138 and W157 are unique to *S. mutans* YidC1 and are present in TM2 and the extramembrane domain that connects TM2 and TM3, respectively. The analogous positions within YidC2 are occupied by Ser and Gly, respectively, both of which contain small side chains and therefore, unlike aromatic residues, would not be expected to contribute to membrane anchoring. The presence of more Trp residues within YidC1 compared to YidC2 suggests that YidC1 is more firmly anchored within the membrane thereby conferring a more rigid structure. Substitution of Trp at position 138 of YidC1, and acquisition of Trp at position 152 of YidC2, would be expected to cause the former to gain flexibility and the latter to lose flexibility within a CL-rich membrane, consistent with their modified functional attributes. In a database analysis of membrane proteins related to human genetic diseases, a random mutation at Trp or Cys was found to have highest probability in translating to a disease state (Vitkup et al., 2003). The role of these residues in the structural stability of the proteins has been proposed to be the reason for the diseased state as a result of mutation. Phospholipid residency maps of WT and mutant proteins in a native-like simulated membrane are expected to provide important insights in this regard too.

Acid- and salt-sensitivity of the *ΔyidC1 yidC2* ^*S152W*^ -expressing strain is similar to that of the *ΔyidC2* mutant that still expresses *yidC1*. Thus, a single amino acid residue at analogous positions within the TM2 helix of YidC1 and YidC2, respectively, confers paralog-specific phenotypic attributes. While the behavior of YidC2^S152W^ and YidC2^S152R^ mutants during growth in excess zinc is not explained by single amino acid substitution, this may stem from as yet unknown changes in cytoplasmic membrane lipid composition during growth in such an environment. The C1 loop of *E. coli* YidC was shown to contact SecY within the holo-translocon (Petriman et al., 2018), in addition to its contact with substrate (Kumazaki et al., 2014a). Our CGMD simulations did not include SecYEG or putative substrates; therefore, phenotypic characteristics of certain YidC2 point mutations are not yet fully understood and remain a future objective. Our previous studies have suggested that YidC1 generally functions as the housekeeping paralog in concert with SecYEG, while YidC2 functions in concert with the SRP pathway under conditions of acid, osmotic, and oxidative stress (Hasona et al., 2005; Lara Vasquez et al., 2021).

Taken together, the results of our CGMD simulations of interactions of *S. mutans* YidC1, YidC2, and variants thereof, with specific lipids within a CL-rich bilayer, coupled with phenotypic characterization of *S. mutans* harboring single YidC1 or YidC2 variants, has substantially advanced our understanding of how these two paralogs accomplish needed insertase activities under varying environmental conditions, and consequently varying membrane lipid compositions. Finally, this study has advanced our understanding of the coordinated roles played by CL and YidC2 in imparting tolerance to acid and osmotic stress, both defining characteristics of this highly resilient oral pathogen.

## Materials and methods

### Bacterial strains, plasmids, and growth conditions

Bacterial strains and plasmids used in this study are listed in Supplementary Table S1. All of the *S. mutans* mutants used in this study were derived from *S. mutans* strain UA159 (Ajdic et al., 2002), and were routinely grown in Todd-Hewitt broth (BBL, Becton Dickinson) supplemented with 0.3% yeast extract (THYE) at 37 °C in 5% CO_2_/95% air atmosphere (v/v). Spectinomycin (1 mg ml^-1^), kanamycin (1 mg ml^-1^), or erythromycin (10 μg ml^-1^) were added, where appropriate, for the growth and selection of *S. mutans* strains. *E. coli* strain C2987 (NEB) was used for genetic manipulations and was routinely grown in Luria–Bertani (LB) medium (10 g l^-1^ tryptone, 5 g l^-1^ yeast extract and 5 g l^−1^ NaCl) at 37°C with vigorous shaking or on LB agar (1.5%) with appropriate selection. Ampicillin (100 μg ml^−1^), or erythromycin (250 μg ml^−1^) were used for the growth and selection of *E. coli* transformants.

Site-directed mutagenesis of *yidC1* and *yidC2*. A standard PCR-based mutagenesis technique was used for site-directed mutagenesis (Zheng et al., 2004). pSM15 and pSM20 (Mishra and Brady, 2021) were used as templates for the mutagenesis of *yidC1* and *yidC2* genes, respectively. Primers used for mutagenesis are listed in Supplementary Table S2.

### Construction of *S. mutans* strains expressing single *yidC1* or *yidC2* variants

*S. mutans* strains were engineered to harbor the *yidC1* or *yidC2* variant of interest expressed under the regulatory control of the respective native promoter. Next, the residual *yidC*1 or *yidC2* gene was deleted by allelic replacement with an antibiotic resistance cassette. Replacement cassettes consisting of the wild-type or mutant *yidC1* or *yidC2* gene were fused to an antibiotic resistance marker gene followed by the 3′-flanking regions of *yidC1* or *yidC2,* respectively. To make the cassettes, PCR amplification products of the three regions were assembled by the NEB HiFi DNA assembly kit following the manufacturer’s instructions. The assembled products served as templates for PCR to generate gene the complete replacement cassettes using end primers. These were then used for transformation of *S. mutans* strain UA159. Once transformants were confirmed to harbor the desired *yidC1* or *yidC2* variant replacements, they were transformed with an allelic replacement construct that comprised the PCR-amplified 5′- and 3′-flanking regions of the residual *yidC* joined to an antibiotic resistance encoding gene. All mutations were confirmed by PCR and sequencing of the relevant genomic loci. Production of YidC1 or YidC2 and their derivatives was confirmed by Western blot using anti-C-terminal tail-specific rabbit antibodies (Mishra and Brady, 2021). Primers used for construction of deletion mutants are listed in Supplementary Table S2.

### Construction of promoter-reporter gene fusions

Promoter-reporter gene fusions were constructed for *yidC1, yidC2,* and *cls.* For the *yidC1* promoter, 470 bps of DNA located directly upstream of the *yidC1* start codon, which included the promoter and partial sequence of *rnpA* was amplified using primers listed in Supplementary Table S2. For the *yidC2* and *cls* promoter-reporter gene fusions, 289 and 300 bp segments upstream of *yidC2* and *wapA* start codons were amplified, respectively. PCR products corresponding to promoters were subcloned between BamHI/SacI digested pMZ plasmid (Chen et al., 2002; Liu et al., 2009), which contains a promoterless *lacZ* gene derived from *Streptococcus salivarius* for integration within *S. mutans* genomic DNA by a double crossover event at *mtlA-phnA* locus. Promoter-lacZ fusion containing plasmids were transformed in *S. mutans* UA159 competent cells and the integrity of the gene fusions was verified by PCR amplification of the relevant locus followed by sequencing.

### β-galactosidase assay

Exponential phase cultures (OD_600_ = 0.2) of promoter-reporter fusion strains grown in THYE were pelleted and resuspended in THYE pH 7.0 (control), THYE pH 5 (acid stress), THYE +3% NaCl (osmotic stress), THYE +2.5 mM ZnCl2 (Zn-excess), THYE +250 μg mL^-1^ bacitracin (cell envelope stress) for 45 min. Pellets were harvested and washed with Z-buffer ((60 mM Na_2_HPO_4_, 40 mM NaH_2_PO_4_, 10 mM KCl, 1 mM MgSO_4_, freshly-added 50 mM β-mercaptoethanol), and β-galactosidase activity was determined and expressed as described previously (Seaton et al., 2015).

### Growth and stress tolerance assay in broth and on agar plates

Overnight cultures grown in THYE broth with appropriate selection agents were diluted 1:50 in THYE and grown to mid-exponential phase (OD_600_ = 0.2–0.4). Following 1:100 dilution of mid-log phase cultures, bacterial growth was measured using a Bioscreen C instrument as described (Mishra and Brady, 2021). Wells of the honeycomb plates used in the Bioscreen C instrument were covered with ∼50 μl of heavy mineral oil to achieve anaerobic growth under non-stress and acid stress conditions, except under conditions of Zn-excess in which mineral oil was not used because zinc toxicity is only observed under aerobic conditions. For efficiency of plating assays, mid-exponential phase cultures were adjusted to OD_600_ = 0.2. Serial ten-fold dilutions were made in THYE, and 4 μL aliquots of each dilution were spotted on THYE agar (pH 7.0, pH 5.0, 3% NaCl, or 2.5 mM ZnCl_2_). Plates were incubated at 37 °C in air with 5% CO_2_ for 2 days before documentation.

### Coarse grain molecular dynamics (CGMD) simulations

Three-dimensional models of the monomeric YidC1 and YidC2 were built using the I-TASSER homology modeling webserver using *B. halodurans* YidC2 (PDB ID: 3WO6) and *E. coli* YidC (PDB ID: 3WVF) as templates (Kumazaki et al., 2014a; Kumazaki et al., 2014b; Yang et al., 2015; Yang and Zhang, 2015) (Figure 1B). Alpha Fold predicted models were similar to those derived by I-TASSER except that YidC1 and YidC2 C-terminal tail structures were not predicted with high confidence by Alpha-Fold. Head-to-head YidC1 or YidC2 dimer files were built by cloning and placing the 180-degree rotated monomer from the original monomer. TM3-4 of each monomer was designated as the interface with a minimum backbone-backbone distance of 7–8 Å at the closest point, measured via Pymol. Monomer and dimer structure files were aligned in a mock membrane using the PPM 2.0 webserver (Lomize et al., 2012). Point mutations were introduced into each WT insertase membrane aligned model structure using the PyMol mutagenesis wizard in order to build mutant systems. Groningen Machine for Chemical Simulation (GROMACS) (Abraham et al., 2015) system files of box size 200 nm, 310.15 K, and with 50 mM NaCl, and 1 bar pressure were built using the CHARMM-GUI (Jo et al., 2008; Lee et al., 2016) Martini maker (Qi et al., 2015; Hsu et al., 2017). Single systems of each protein, monomer or dimer, were built into lipid mixtures containing either 60 mol% CL or PE, with 20 mol% PI and 20 mol% PS. Lipid acyl chains were 1-palmitoyl-2-oleoyl (PO) in all cases. A schematic diagram of phospholipid molecules employed in this study is shown in Supplementary Figure S1. Each system was energy minimized and equilibrated according to default CHARMM-GUI mdp settings with the Martini2.2 forcefield (Monticelli et al., 2008; Periole et al., 2009; d et al., 2013), using the Berendensen Barostat (Berendsen et al., 1984) v-rescale thermostat, and the reaction-field method of electrostatics (Hünenburger and Gunsteren, 1998). After equilibration, each system was replicated for production to ensure equivalent starting energy profiles for a total of 5 replicates per system (Knapp et al., 2018). Production was carried out for 10 µs with identical parameters with the exception of the Parrinello-Rahman Barostat (Parrinello and Rahman, 1981) in place of the Berendensen Barostat. Production mdp files were altered only to influence simulation time length. Computational time was provided by the Texas Tech High Performance Computing Center (HPCC). Protein Cα root-mean-square deviation (RMSD) analysis was performed with the gmx rms tool and calculated as average Cα RMSD as a function of simulation time compared to the final structural snapshot from equilibration trajectories per simulation. RMSD is presented as mean ± s.d. from the 5 replicate systems. Average root-mean-square fluctuations (RMSF) per residue were calculated using the gmx rmsf tool. RMSF data is presented as mean ± s.d. of each residue throughout simulation across 5 replicates. Two-tailed t-tests from RMSF data were calculated between per replicate residue RMSF values across datasets. Lipid residency times were calculated using PyLipID (Song et al., 2022) with an interaction start distance of 0.55 nm and an end distance of 1.0 nm. Data are presented as the mean of 5 replicates.

## Data Availability

The original contributions presented in the study are included in the article/Supplementary Material, further inquiries can be directed to the corresponding author.
