# Supplementary material for: Cardiolipin occupancy profiles of YidC paralogs reveal the significance of respective TM2 helix residues in determining paralog-specific phenotypes
Source: Front Mol Biosci. 2023 Oct 6;10:1264454. doi: 10.3389/fmolb.2023.1264454 (PMC10588454; doi:10.3389/fmolb.2023.1264454)
Supplement: Supplementary file 1 [file DataSheet2.PDF]

Table S1. List of bacterial strains and plasmids used in this study.

**Strains**	**Genotype/description**	**Source/reference**
Streptococcus mutans		
UA159	Wild-type (serotype c)	[52]
SM2336	UA159 *ΔyidC1(46-666)::*aad9; Spc^R	This work
SM2670	UA159 *ΔyidC2::ermB*; Erm^R	This work
SM2733	UA159 *yidC1*^{W138A} *ΔyidC2::ermB*; Spc^R, Erm^R	This work
SM2800	UA159 *yidC1*^{W138R} *ΔyidC2::ermB*; Spc^R, Erm^R	This work
SM2694	UA159 *yidC2*^{S152A} *ΔyidC1::aad9*; Spc^R, Erm^R	This work
SM2697	UA159 *yidC2*^{S152R} *ΔyidC1::aad9*; Spc^R, Erm^R	This work
SM2703	UA159 *yidC2*^{S152W} *ΔyidC1::aad9*; Spc^R, Erm^R	This work
SM2401	UA159 PyidC1::lacZ; Kan^R	This work
SM2259	UA159 PyidC2::lacZ; Kan^R	This work
SM2571	UA159 Pcls::lacZ; Kan^R	This work
Escherichia coli		

C2987	*fhuA2 Δ(argF-lacZ)U169 phoA glnV44 f80Δ(lacZ)M15 gyrA96 recA1 relA1 endA1 thi-1 hsdR17*	NEB
**Plasmids**		
pSM528	pET15b-ΔyidC1::aad9; Amp^R	This work
pSM525	pPMZ-PyidC1; Kan^R	This work
pSM331	pPMZ-PyidC2; Kan^R	This work
pSM765	pPMZ-Pcls; Kan^R	This work
pSM15	pETDuet1-yidC1; Amp^R	[11]
pSM20	pET15b-yidC2; Amp^R	[11]
pSM244	pETDuet1-yidC1W138A; Amp^R	This work
pSM106	pETDuet1-yidC1W138R; Amp^R	This work
pSM217	pET15b-yidC2S152A; Amp^R	This work
pSM214	pET15b-yidC2S152R; Amp^R	This work
pSM305	pET15b-yidC2S152W; Amp^R	This work

Table S2: List of primers

Name	Sequence (5'→3')	Restriction enzyme	Direction	Complementary region	Comments
SM306	ctggtgccgcgcggcagccaTGGCTGATTATTTAGCCAG	None	Fwd	+1121 to +1145 bp of smu_335.	For amplification of 1st fragment of *ΔyidC1::aad9* cassette.
SM440	ccatagttgtAAGTGCTGCAACAGCTAATC	None	Rev	+45 to +26 bp of *yidC1*.	
SM441	tgcagcacttACAACTATGGATATAAAATAGGTACTAATC	None	Fwd	-78 to -49 of *aad9* (pDL278)	For amplification of 2nd fragment of *ΔyidC1::aad9* cassette.
SM442	catttgagacGTTTCCACCATTTTTTCAATTTTTTATAATTTTTTAATC	None	Rev	+710 to +750 of *aad9* (pDL278 plasmid)	
SM443	tggtggaaacGTCTCAAATGCTTATCAGGTC	None	Fwd	+667 to +687 bp of *yidC1*.	

SM311	cgggctttgttagcagccgATTGCAATGATTTTAAAACCTTG	None	Rev	+666 to +688 bp of jag.	For amplification of 3rd fragment of *ΔyidC1::aad9* cassette.
SM489	gtttttcttttgGATAATCTTCCTTTGATTTTAATAATAC	None	Rev	-28 to -1 bp of yidC2.	Amplification of yidC2 5'flank with ermB overhang. 1st fragment of DyidC2-ermB.
SM481	ACAAAAACAGCATCAAAAG	None	Fwd	+380 to +398 bp of smu_1724c.	
SM490	aaggaagattatcCAAAAGAAAAACGAAATGATACAC	None	Fwd	-261 to -238 bp of ermB (pMSP3535)	PCR amplification of ermB cassette.
SM491	tataaaattagcTTATTTCCTCCCGTTAAATAATAG	None	Rev	+715 to +738 bp of ermB.	
SM492	cgggaggaaataaGCTAATTTTATACTCATAGACTG	None	Fwd	+934 to +956 bp of yidC2.	PCR amplification of 700 bp 3'-flank of yidC2.
SM493	AACGTATTAAAATAGCACGTTC	None	Rev	+365 to +386 bp of greA.	

SM273	CGTTCTTGCTGCTCTCTATCAAGCCCTTAC	None	Fwd	+404 to +434 bp of yidC1W138A	PCR mutagenesis of yidC1 to yidC1W138A.
SM274	TAGAGAGCAGCAAGAACGGGCATCTGAATAAG	None	Rev	+391 to +422 bp of yidC1W138A	
SM185	GCCCGTTCTTcgtGCTCTCTATC	None	Fwd	+402 to +424 bp of yidC1	PCR mutagenesis of yidC1W138R using pSM15 template.
SM186	ATCTGAATAAGAAGAGGAAAAAG	None	Rev	+379 to +401 bp of yidC1.	
SM259	TTTCTTCGCTGCACTTTATATTTCTACCCGTTA	None	Fwd	+447 to +479 bp of yidC2 S152A	PCR mutagenesis of yidC2 to yidC2S152A
SM260	TAAAGTGCAGCGAAGAAAGGCATCTGGATCAG	None	Rev	+433 to +464 bp of yidC2 S152A	
SM261	TTTCTTCCGTGCACTTTATATTTCTACCCGTTA	None	Fwd	+447 to +479 bp of yidC2 S152R fwd	PCR mutagenesis of yidC2 to yidC2S152R.

SM262	TAAAGTGCACGGAAGAAAGGCATCTGGATCAG	None	Rev	+433 to +464 bp of yidC2 S152R	
SM253	TTTCTTCTGGGCACTTTATATTTCTACCCGTTA	None	Fwd	+447 to +479 bp of yidC2 S152W	PCR mutagenesis of yidC2 to yidC2S152W
SM254	TAAAGTGCCCAGAAGAAAGGCATCTGGATCAG	None	Rev	+433 to +464 bp of yidC2 S152W	
SM400	GAATTGGAGCTCGATATTTATGGACGTGTTTGGAAC	SacI	Fwd	+88 to +111 of smu_1725.	PCR amplification of 283 bp *yidC2* promoter sequence. Tm = 58º C (Q5) and 45º C (one taq).
SM401	GCGGGATCCGATAATCTTCCTTTGATTTTAATAATACC	BamHI	Rev	-1 to -29 bp of *yidC2*.	
SM702	GAATTGGAGCTCCTTTAAGATTTATGGTGTTGCTTGTTG	SacI	Fwd	-242 to -216 bp of wapA (intergenic region).	PCR amplification of *wapA* promoter region that also regulates *clsA* for cloning in pPMZ.

SM703	GCGGGATCCAATAGAATTTTCTCCTTAGTTAATCTGTAG	BamHI	Rev	-30 to -1 bp (intergenic region).	
JM1	GAATTGGAGCTCCACTTAAATCACATACGGCCG	SacI	Fwd	-195 bp to -174 bp of rnpA	PCR amplification of *yidC1* promoter region for cloning in pPMZ
SM988	GCGGGATCCGAATAGATCCTTCCTGATACAGTTTA	BamHI	Rev	+318 to +343 bp of rnpA.	
