# Supplementary material for: Cardiolipin occupancy profiles of YidC paralogs reveal the significance of respective TM2 helix residues in determining paralog-specific phenotypes
Source: Front Mol Biosci. 2023 Oct 6;10:1264454. doi: 10.3389/fmolb.2023.1264454 (PMC10588454; doi:10.3389/fmolb.2023.1264454)
Supplement: Supplementary file 2 [file DataSheet1.PDF]

Fig. S1

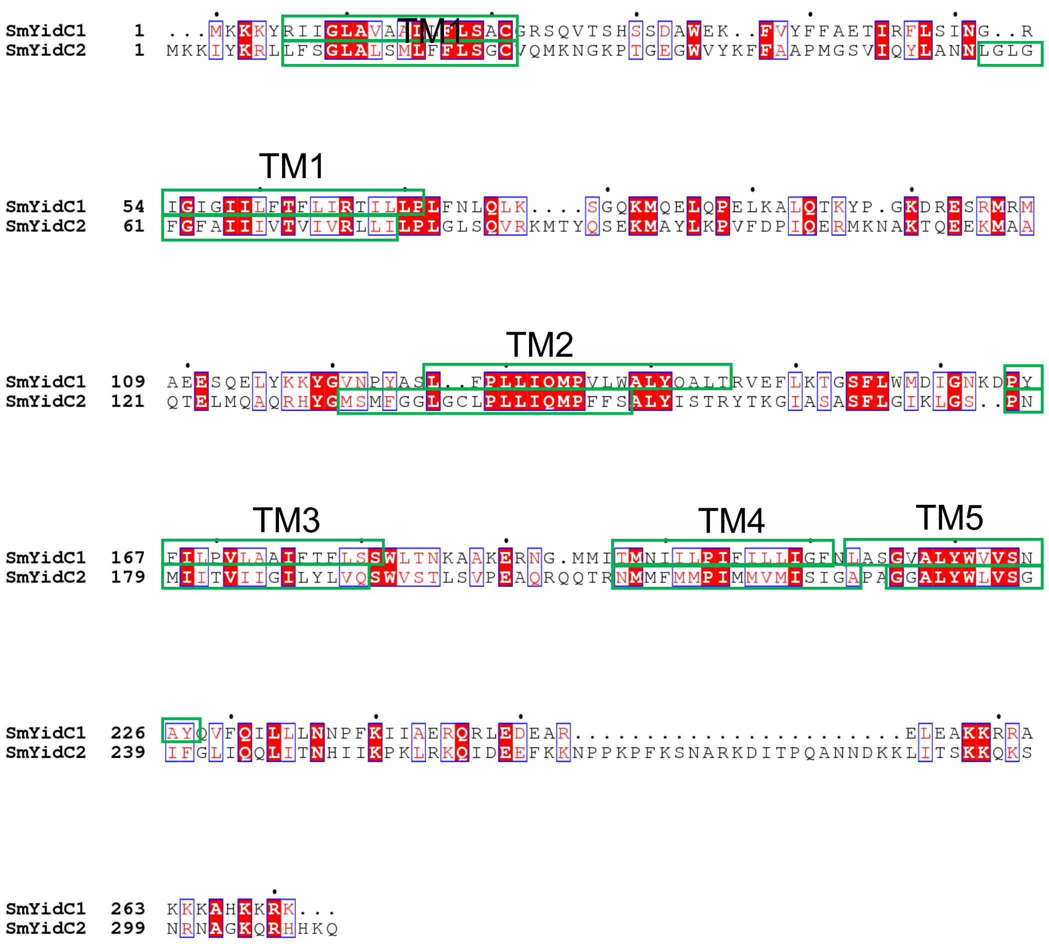

Fig. S1. Amino acid sequences of *S. mutans* YidC1 and YidC2 aligned by Clustal W presented using the ESPript3.0 program (https://espript.ibcp.fr) (Robert and Gouet, 2014)**.**

Fig. S2

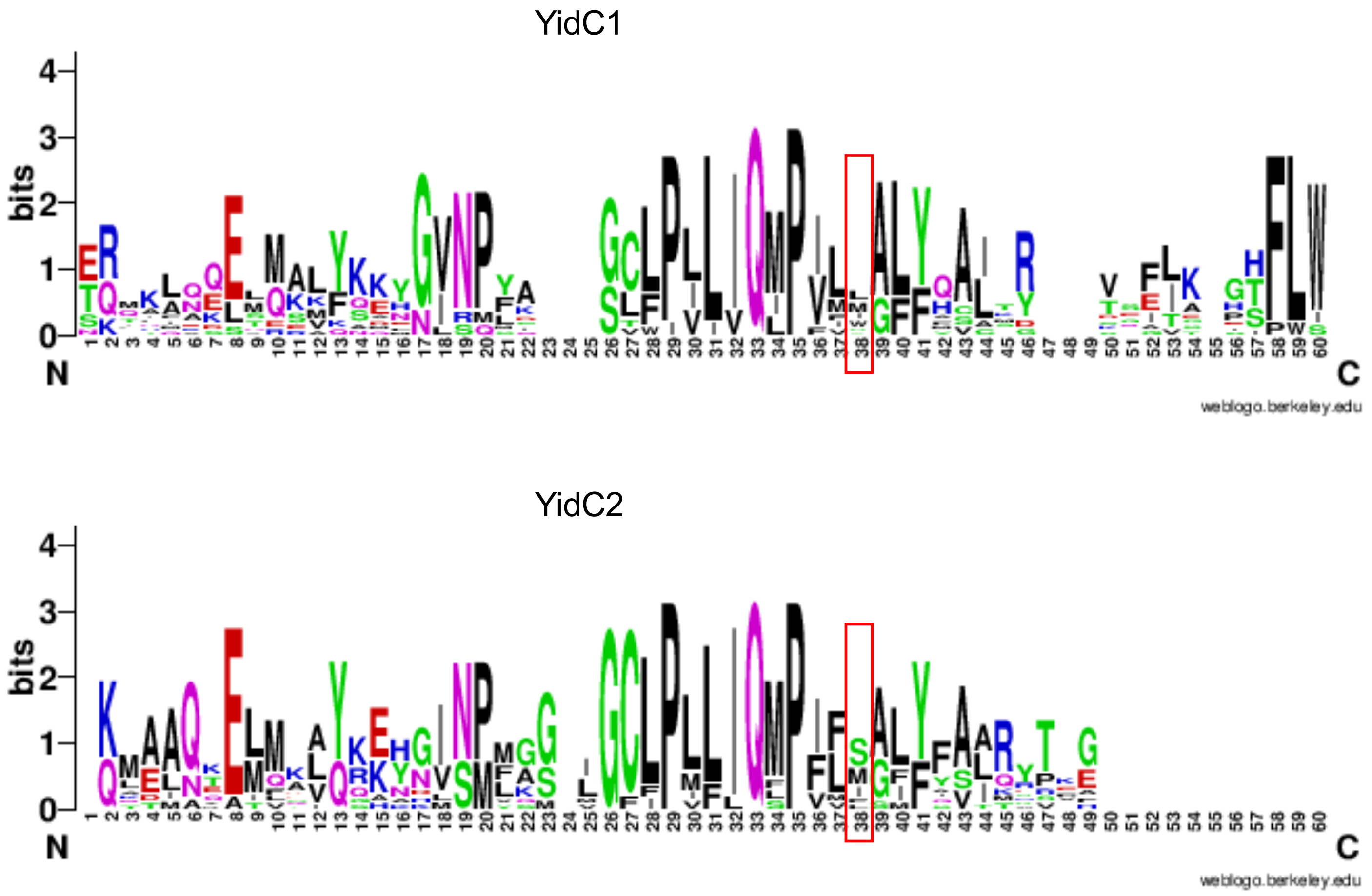

Fig. S2: Sequencelogo representing conservation of residues in YidC1/2 of Gram-positive bacteria. The conservation of the residues corresponds to the height of the letter and the sequence logo was generated using the program WebLogo.

Fig. S3

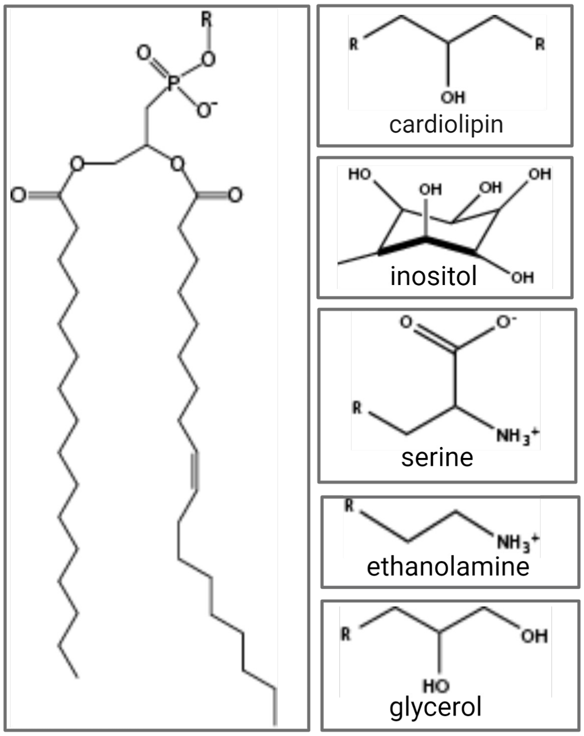

Fig. S3. Structures of lipids evaluated in the current study.

Fig. S4

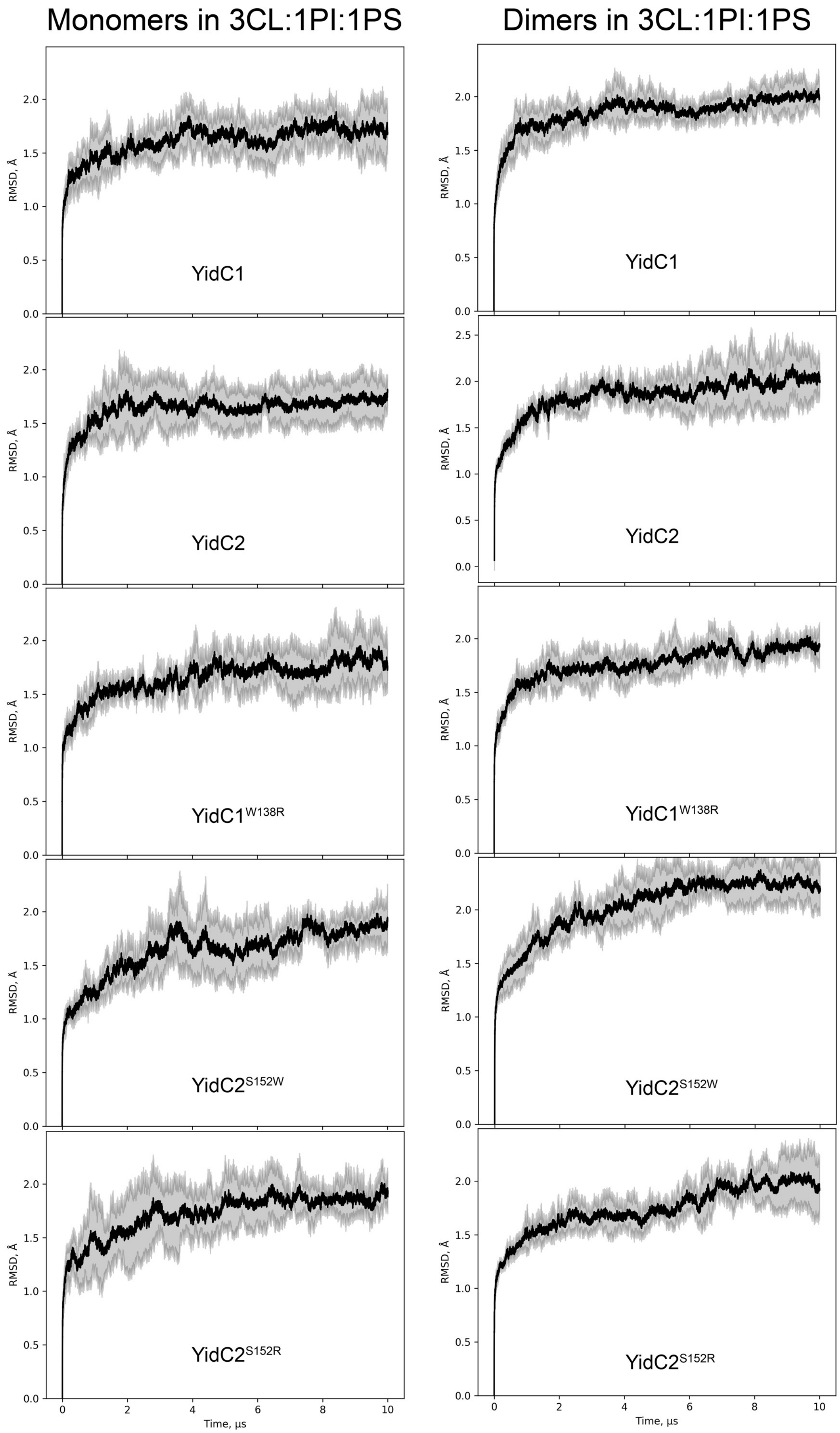

Fig. S4. Root-mean-square deviations (RMSD) of monomeric and dimeric structures of YidC1, YidC2, or indicated modified proteins, during coarse grain molecular dynamics experiments in simulated tri-lipid mixtures. Data are presented as the mean ± s.d. of 5 independent replicates.

Fig. S5

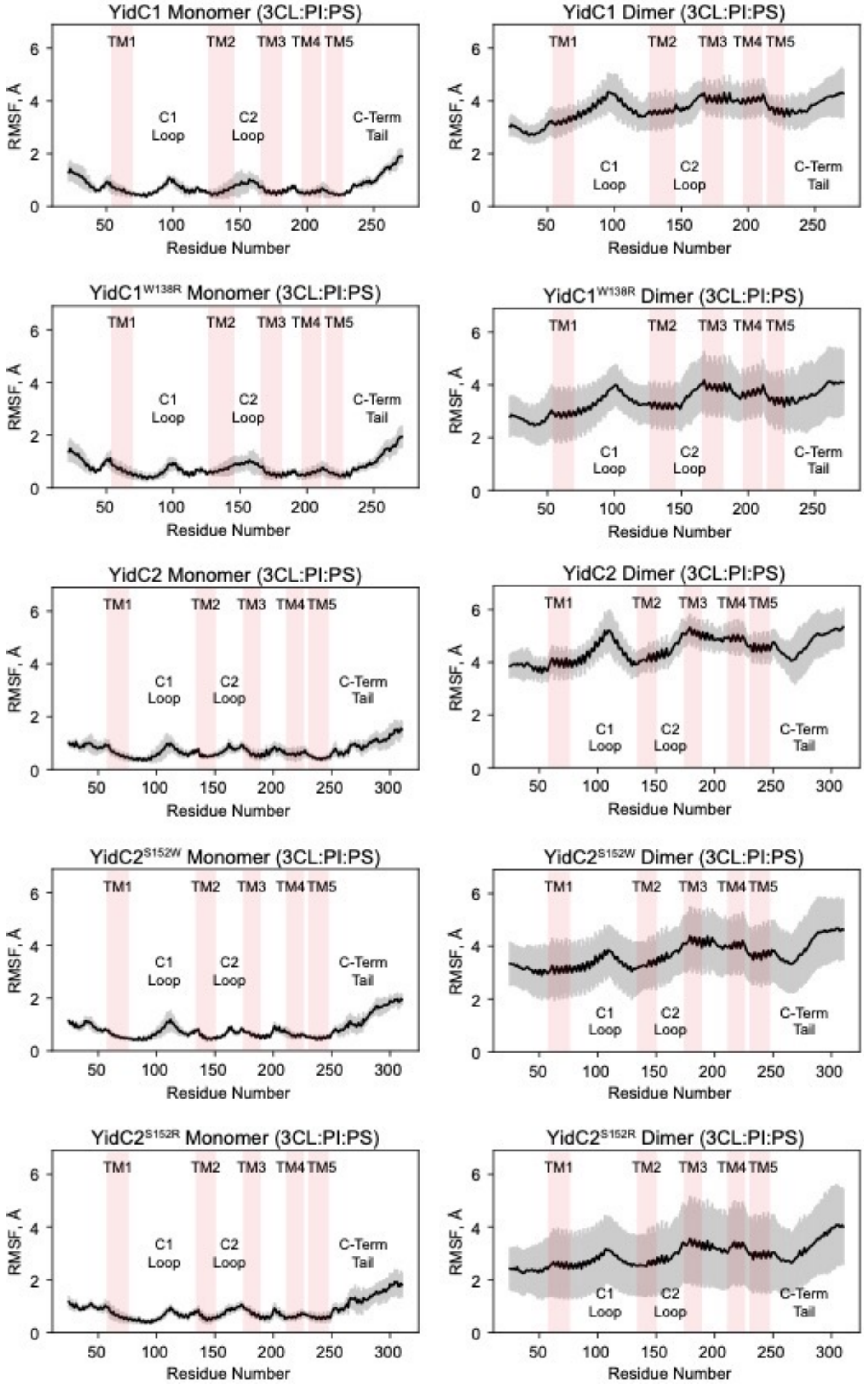

Fig. S5. Root-mean-square fluctuations (RMSF) of individual residues of monomeric or dimeric YidC1, YidC2, or indicated modified proteins, during coarse grain molecular dynamics experiments in simulated tri-lipid mixtures. Pink shading indicates residues comprising transmembrane domains 1 through 5. Locations of the C1 and C2 cytoplasmic loops and the C-terminal cytoplasmic tail segments are also indicated. Data are presented as the mean ± s.d. of 5 independent replicates.

Fig. S6

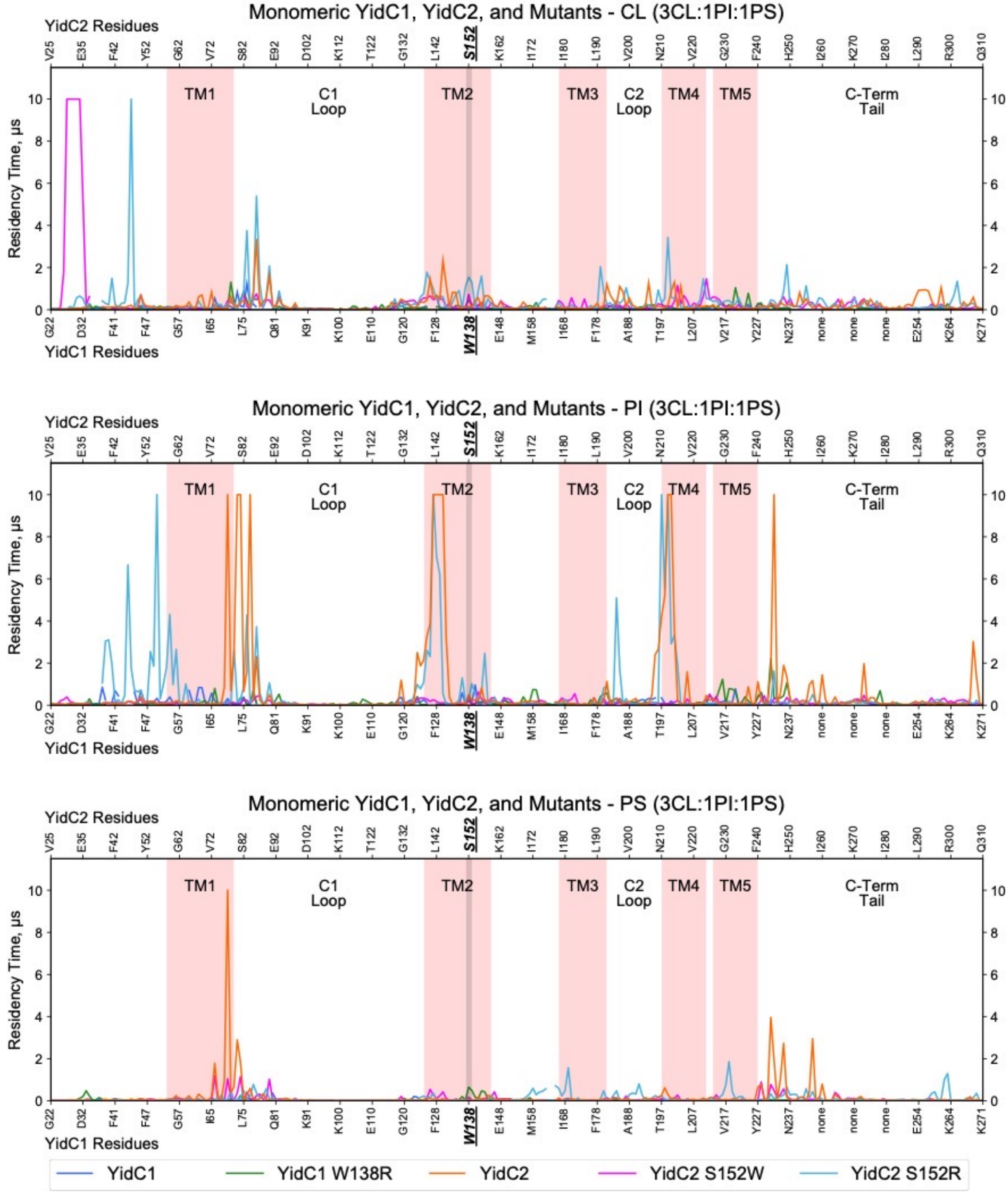

Fig. S6. Comparison of CL, PI, and PS phospholipid occupancy of various YidC1 and YidC2 monomeric variants derived from CGMD in a simulated CL-rich bilayer. Residency times of individual numbered residues of YidC1 (denoted at the bottom of each graph) or YidC2 (denoted at the top of each graph) are shown. Pink shading indicates residues comprising transmembrane domains 1 through 5. Locations of the C1 and C2 cytoplasmic loops and the C-terminal cytoplasmic tail segments are also indicated. Location of W138/S152 residue is highlighted in grey.

Fig. S7

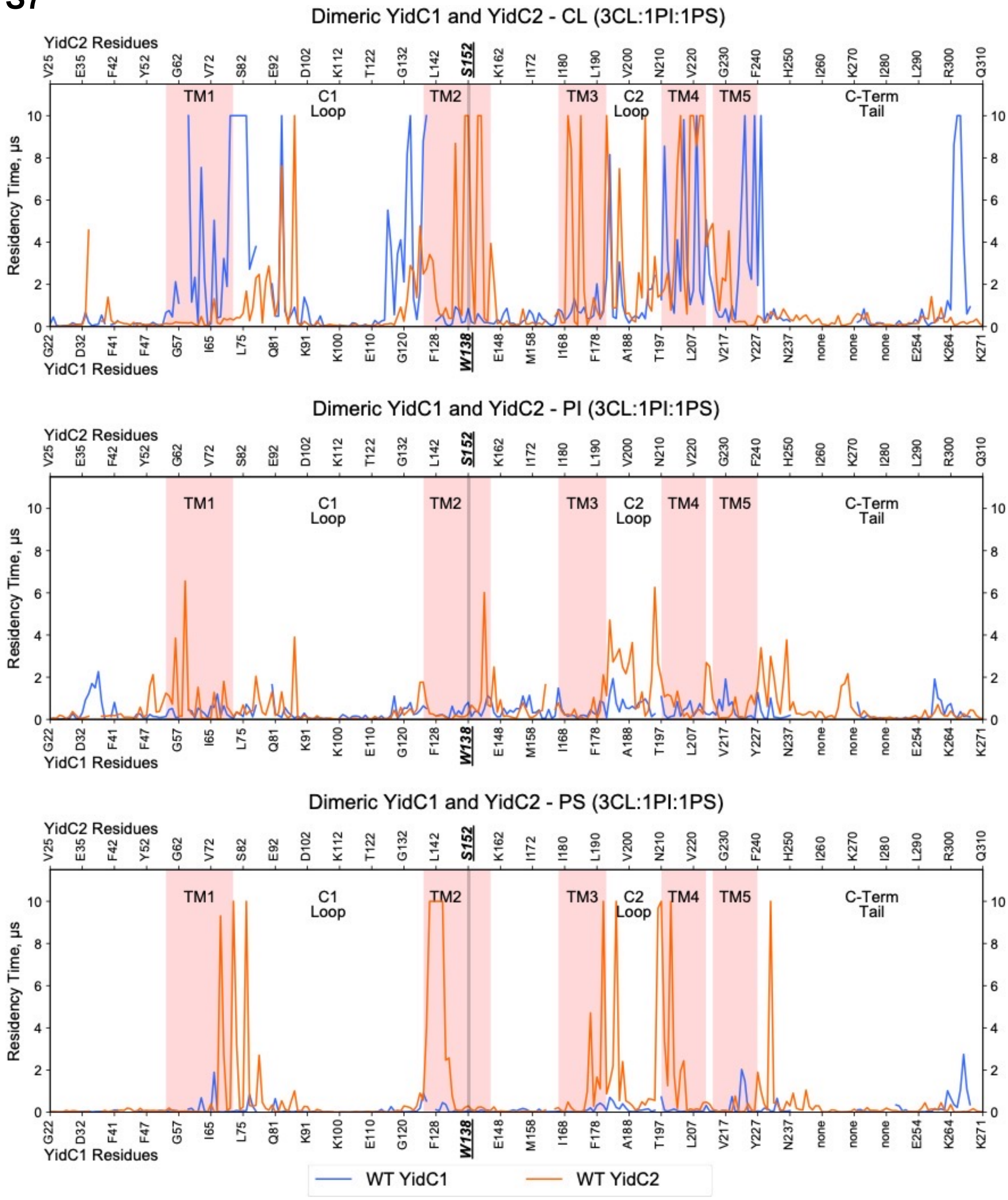

Fig. S7. Comparison of CL, PI, and PS phospholipid occupancy of dimeric YidC1 and YidC2 derived from CGMD in a simulated CL-rich bilayer. Residency times of individual numbered residues of YidC1 (denoted at the bottom of each graph) or YidC2 (denoted at the top of each graph) are shown. Pink shading indicates residues comprising transmembrane domains 1 through 5. Locations of the C1 and C2 cytoplasmic loops and the C-terminal cytoplasmic tail segments are also indicated. Location of W138/S152 residue is highlighted in grey.

Fig. S8

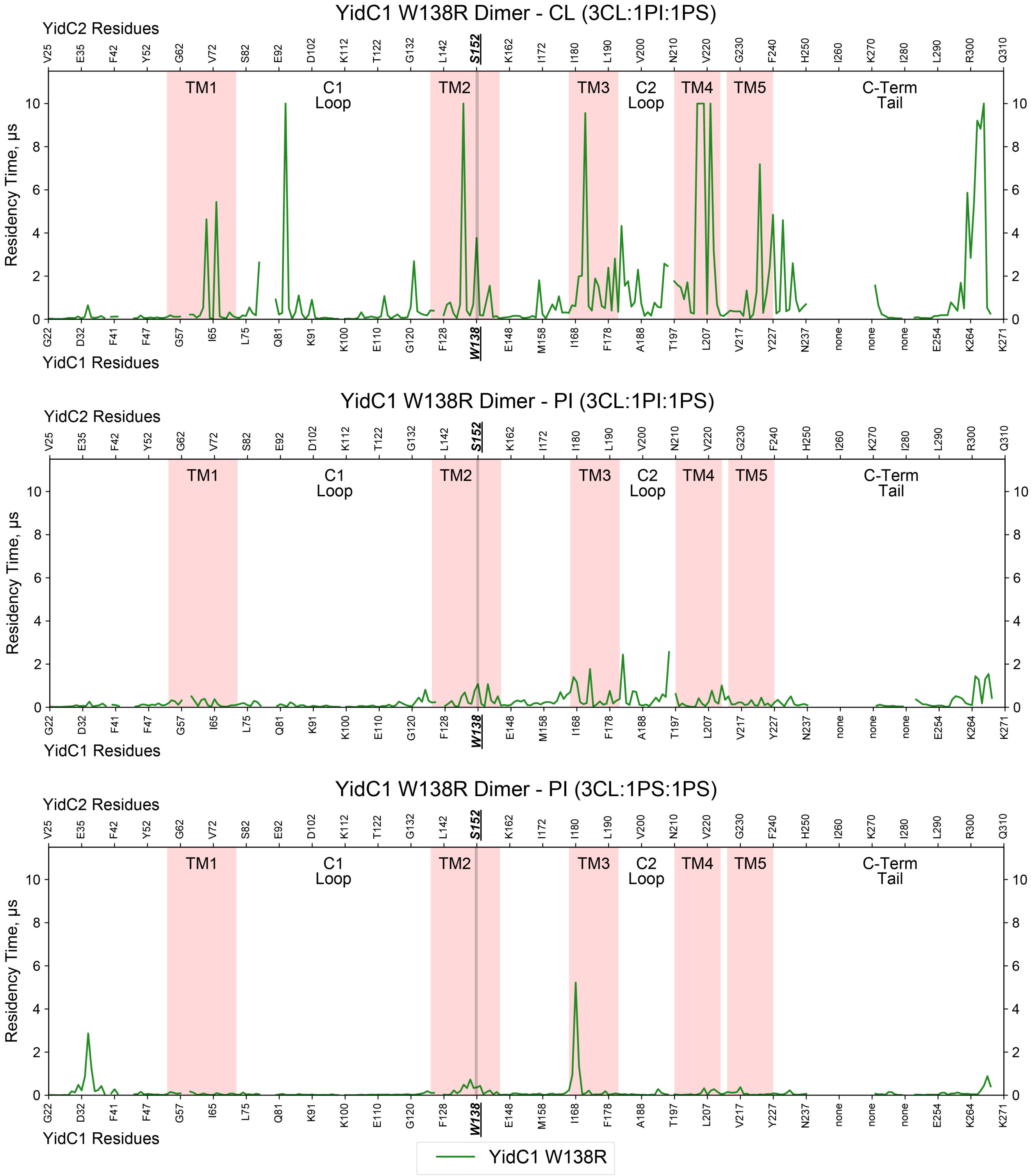

Fig. S8. CL, PI, and PS phospholipid occupancy of dimeric YidC1W138R derived by CGMD in a simulated CL-rich bilayer. Residency times of individual numbered residues of YidC1 (denoted at the bottom of each graph) or YidC2 (denoted at the top of each graph) are shown. Pink shading indicates residues comprising transmembrane domains 1 through 5. Locations of the C1 and C2 cytoplasmic loops and the C-terminal cytoplasmic tail segments are also indicated. Location of W138/S152 residue is highlighted in grey.

Fig. S9

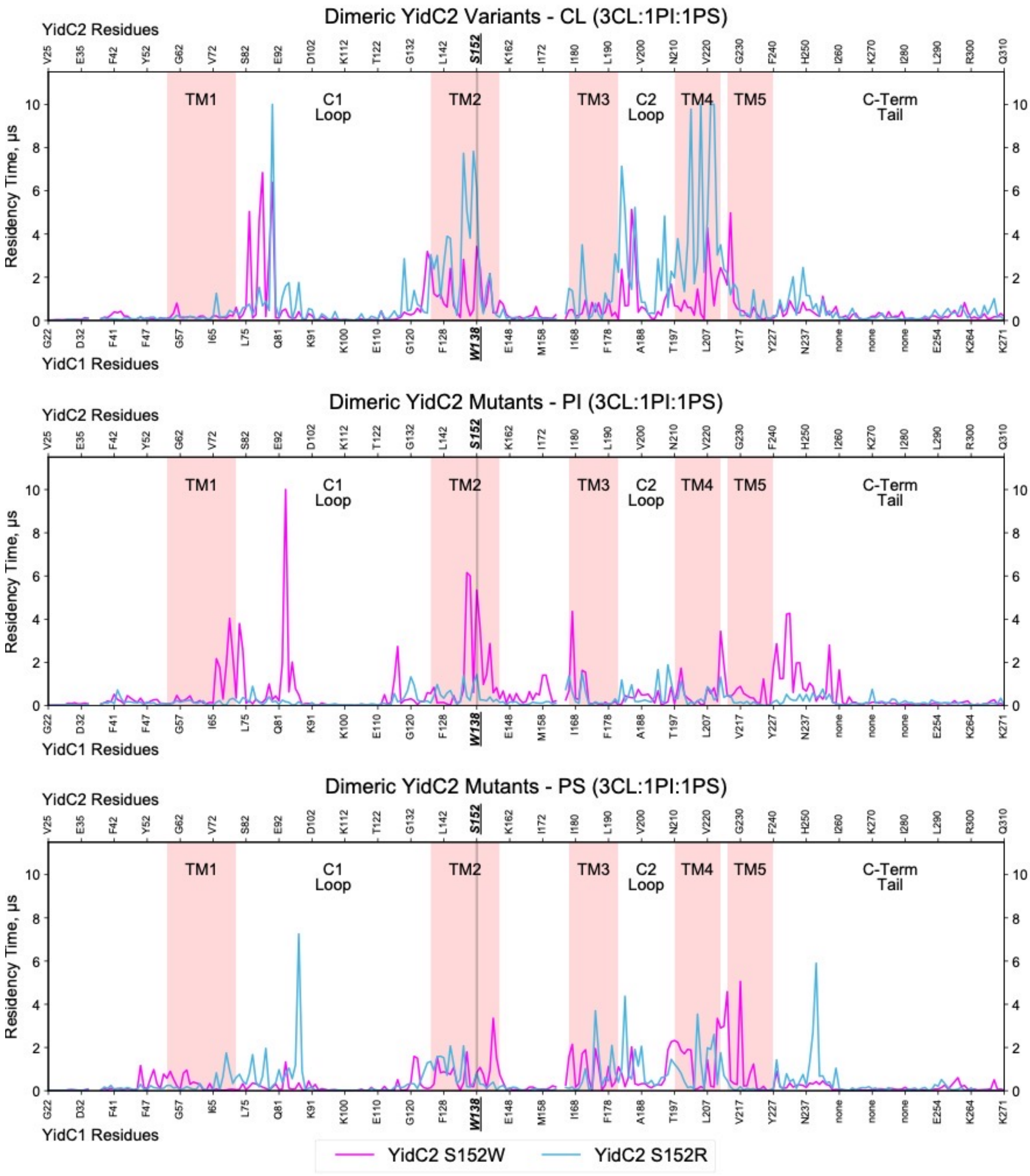

Fig. S9. CL, PI, and PS phospholipid occupancy of dimeric YidC2 variants derived by CGMD in a simulated CL-rich bilayer. Residency times of individual numbered residues of YidC1 (denoted at the bottom of each graph) or YidC2 (denoted at the top of each graph) are shown. Pink shading indicates residues comprising transmembrane domains 1 through 5. Locations of the C1 and C2 cytoplasmic loops and the C-terminal cytoplasmic tail segments are also indicated. Location of W138/S152 residue is highlighted in grey.
